# Supplementary material for: Mass spectrometric based detection of protein nucleotidylation in the RNA polymerase of SARS-CoV-2
Source: Commun Chem. 2021 Mar 19;4:41. doi: 10.1038/s42004-021-00476-4 (PMC8238455; doi:10.1038/s42004-021-00476-4)
Supplement: Supplementary file 1 — Supplementary Information [file 42004_2021_476_MOESM1_ESM.pdf]

Supplementary Information

Mass spectrometric based detection of protein nucleotidylation in the RNA polymerase of SARS-CoV-2

Brian J. Conti[1,2], Andrew S. Leicht [1,3], Robert N. Kirchdoerfer[1,3] and Michael R. Sussman[1,2,*]

[1] Department of Biochemistry, University of Wisconsin-Madison, Madison, WI 53706

[2] Center for Genomic Science Innovation, University of Wisconsin-Madison, Madison, WI 53706

[3] Institute for Molecular Virology, University of Wisconsin-Madison, Madison, WI 53706

*To whom correspondence should be addressed: msussman@wisc.edu

Supplementary Methods

Nidovirus sequence alignment and conservation. To examine conservation of GMP-modification sites, the SARS-CoV-2 and EAV polyprotein 1ab Genbank sequences were compared those encoded by the same *Nidovirales* order representative species examined in Lehmann *et al.* [1], in addition to SARS-CoV-2. These represented the *Arteriviridae*, *Coronaviridae*, *Mesoniviridae* and *Ronivirida* families in the *Nidovirales* order. The Porcine reproductive and respiratory syndrome virus (PVVR2) with accession JX138233 could not be found in Genbank, and therefore, five alternate strains of the virus were used instead. The full list of *Nidovirales* species is as follows ((Genbank accession number) (protein name) [species]):

1) YP_001661452.1 ORF1ab replicase polyprotein pp1ab [*Gill-associated virus*]
2) YP_009666324.1 replicase polyprotein 1ab [*Yellow head virus*]
3) AEH26445.1 replicase polyprotein 1ab [*Cavally virus*]
4) YP_009026378.1 ORF 1ab [*Casuarina virus*]
5) BAN58307.2 pp1a polyprotein [*Dak Nong virus*]
6) YP_007697629.1 ORF1ab [*Hana virus*]
7) YP_007697642.1 ORF1ab [*Nse virus*]
8) YP_007697636.1 ORF1ab [*Meno virus*]
9) YP_008798230.1 polyprotein 1ab [*Porcine torovirus*]
10) YP_009052475.1 pp1ab [*Ball python nidovirus 1*]
11) YP_337905.2 polyprotein 1ab [*Breda virus*]
12) ABI97394.1 replicase polyprotein 1ab [*White bream virus*]
13) YP_009505581.1 replicase polyprotein 1ab [*Fathead minnow nidovirus*]
14) AEU12347.3 replicase polyprotein 1ab [*Wobbly possum disease virus*]
15) YP_009109556.3 viral nonstructural polyprotein [*Simian hemorrhagic fever virus*]
16) AZT89154.1 ORF1ab protein [*Simian hemorrhagic fever virus*]
17) AGA19089.1 ORF1a [*Simian hemorrhagic fever virus*]
18) AGA19090.1 ORF1b [*Simian hemorrhagic fever virus*]
19) AEC48046.1 polyprotein [*Kibale red colobus virus 1*]
20) AEC48047.1 polyprotein, partial [*Kibale red colobus virus 1*]
21) AEJ54657.1 replicase polyprotein 1a [*Porcine reproductive and respiratory syndrome virus*]
22) AEJ54658.1 replicase polyprotein 1b, partial [*Porcine reproductive and respiratory syndrome virus*]
23) QPK93580.1 |polyprotein 1ab [*Porcine reproductive and respiratory syndrome virus*]
24) APU51031.1 |polyprotein 1ab [*Porcine reproductive and respiratory syndrome virus*]
25) ANT45956.1 |polyprotein 1ab [*Porcine reproductive and respiratory syndrome virus*]
26) ALL55209.1 |polyprotein 1ab [*Porcine reproductive and respiratory syndrome virus*]
27) AFP43966.1 |polyprotein 1ab [*Porcine reproductive and respiratory syndrome virus*]
28) AAA74103.1 polyprotein [*Lactate dehydrogenase-elevating virus*]
29) AAA74104.1 polyprotein, partial [*Lactate dehydrogenase-elevating virus*]
30) AAA85663.1 polyprotein 1a [*Lactate dehydrogenase-elevating virus*]
31) AAA85664.1 polyprotein 1b, partial [*Lactate dehydrogenase-elevating virus*]
32) YP_001008394.2 polyprotein 1ab [*Lactate dehydrogenase-elevating virus*]
33) ABI64071.1 replicase polyprotein, partial [*Equine arteritis virus*]
34) ABI64079.1 replicase polyprotein, partial [*Equine arteritis virus*]
35) AAP50483.1 putative ORF1ab polyprotein [*SARS coronavirus FRA*]
36) AFE48810.1 polyprotein [*Rabbit coronavirus HKU14*]
37) AAF19383.1 RNA-directed RNA polymerase [*Murine hepatitis virus strain 2*]
38) AAF19384.1 RNA-directed RNA polymerase [*Murine hepatitis virus strain 2*]

39) AAX76519.1 orf1ab polyprotein [*Human coronavirus HKU1*]
40) YP_009513008.1 ORF1ab polyprotein [*Betacoronavirus Erinaceus/VMC/DEU/2012*]
41) ABG47051.1 ORF1 [*Bat coronavirus (BtCoV/133/2005)*]
42) ABN10874.1 orf1ab polyprotein [*Bat coronavirus HKU5-1*]
43) YP_009047202.1 1AB polyprotein [*Middle East respiratory syndrome-related coronavirus*]
44) ADM33581.1 orf1ab polyprotein [*Bat coronavirus HKU9-10-2*]
45) YP_008439200.1 replicase polyprotein 1ab [*Bat coronavirus CDPHE15/USA/2006*]
46) YP_003766.2 replicase polyprotein 1ab [*Human coronavirus NL63*]
47) YP_001718610.1 ORF1ab polyprotein [*Miniopterus bat coronavirus HKU8*]
48) YP_001552234.1 orf1ab polyprotein [*Rhinolophus bat coronavirus HKU2*]
49) YP_001718603.1 ORF1ab polyprotein [*Bat coronavirus 1A*]
50) AFI49429.1 replicase polyprotein 1ab [*Alpaca respiratory coronavirus*]
51) YP_001351683.1 ORF1 [*Scotophilus bat coronavirus 512*]
52) AGK89913.1 polyprotein [*Porcine epidemic diarrhea virus*]
53) AFU92112.1 orf1ab polyprotein [*Rousettus bat coronavirus HKU10*]
54) YP_009019180.1 orf1ab polyprotein [*Mink coronavirus strain WD1127*]
55) ACT10947.1 orf1ab polyprotein [*Feline coronavirus UU2*]
56) AGK85497.1 polyprotein 1ab [*Infectious bronchitis virus*]
57) AHB63480.1 replicase polyprotein [*Bottlenose dolphin coronavirus HKU22*]
58) YP_005352845.1 replicase polyprotein [*Sparrow coronavirus HKU17*]
59) YP_002308505.1 orf1ab polyprotein [*Munia coronavirus HKU13-3514*]
60) YP_005352880.1 orf1ab gene product [*Common moorhen coronavirus HKU21*]
61) YP_002308478.1 orf1ab polyprotein [*Bulbul coronavirus HKU11-934*]
62) YP_009724389.1 ORF1ab polyprotein [*Severe acute respiratory syndrome coronavirus 2*]

These polyprotein 1ab or orf (open reading frame) 1ab proteins encode the large polypeptide that is cleaved by peptidases into the individual viral non-strucutral proteins (nsp) post-translationally (reviewed in Posthuma *et al*.) [2], thus protein accession numbers for single nsp proteins in each species were not available. Some polyprotein Genbank entries were split into orf 1a and orf 1b, thus there are two separate entries for a single species.

Each sequence modified with GMP in this study (EAV nsp9, EAV nsp7, SARS-CoV-2 nsp7) was first aligned against each Nidoviral polyprotein sequences individually using the BLAST algorithm [3,4] in the stand alone BLAST+ software suite (version 2.11.00) available from the National Center of Biotechnology Information at https://ftp.ncbi.nlm.nih.gov/blast/executables/blast+/LATEST/. Genbank polyprotein sequences that did not contain homologous regions with the queried protein were apparent from the alignment results. These sequences were removed, and a multiple sequence alignment was performed on the remaining sequences using the Clustal Omega algorithm via the web interface found at https://www.ebi.ac.uk/Tools/msa/clustalo/ [5].

The SARS-CoV-2 nsp7 sequence was only found in the *Coronaviridae* family, but was strongly conserved [6] as illustrated in Supplementary Figure 18. EAV nsp9 contains the RNA polymerase domain that is conserved throughout the *Nidovirales* order, thus the SARS-CoV-2 nsp12 sequence was included as an addition separate entry in the multiple sequence alignment for clarity [2]. This sequence alignment is found as Supplementary Figure 19. Because the GMP modified residue in EAV nsp9, K380, was completely conserved, a large number of sequences aligned. The SARS-CoV-2 residue K545, that is homologous to EAV nsp9 residue K380, was illustrated in the solved SARS-CoV-2 nsp12/nsp7/nsp8 structure (PDB 7btf) using Pymol software (Supplementary Figure 20) [7]. EAV nsp7 was only found in the *Arteriviridae* family, which has limited number of members. Within this family, the nsp7α portion of the protein is conserved to a stronger extent than nsp7β, which contains the three GMP-modification sites [8-10] (Supplementary Figure 21).

LC-MS/MS strategy for identification of GMP-modified peptide in mutant proteins. Parallel WT GMP-labeled samples were always prepared with the mutant protein versions in the same experiment to ascertain GMP-modification of the original WT sites were observed. LC-MS/MS was acquired both in data-dependent and targeted acquisition modes. Peptide masses and m/z values (for charge states 1 -5) were calculated for unmodified and GMP-modified versions of at least one peptide containing a mutated modification site (See Supplementary Figure 11 and 15 for these peptides). These peptides were expected to have either similar charge states or one less charge compared to the corresponding WT peptide. The predicted m/z of unmodified and GMP-modified peptides were targeted, resulting in acquisition of HCD and EThcD fragmented MS/MS spectra for these predicted m/z values every second of the LC elution gradient. Data was first searched with Proteome Discoverer for MS/MS matches. In all cases, the unmodified, mutant versions of the peptide were readily identified. If a GMP-modified version was not identified by MS/MS searches, the raw data was examined closely. First, we searched for an LC peak that had any of the predicted m/z values and

the correct charge by extracting ion chromatograms for those m/z values from the raw data using Freestyle software (Thermo). Second, we asked if any LC peaks with the predicted m/z value and charge yielded an HCD fragmented spectra containing the diagnostic guanine fragment ion using Freestyle. Only in the instance of the SARS-CoV-2 nsp7 mutant peptide 1-GSAMSDVKCTSVVL-14 were we able to identify the GMP-modified precursor after failing to obtain a MS/MS peptide spectrum match for the peptide. For the EAV nsp7 K156 GMP modification site, we originally discovered the modification on the tryptic peptide 144-GAQLEWDRHQEEKR-157. However, for unknown reasons, both the unmodified and GMP-modified versions of this peptide were absent in LC-MS/MS experiments examining the WT and mutant proteins. Therefore, we examined the K156 GMP-modification site in EAV nsp7 WT and EAV nsp7 K156A in the 150-DRHQEEKRNAGDDDFAVSNDY-170 peptide that was a result of a chymotrypsin digest. The GMP-modification was readily observed in the WT peptide and assigned to K156. The GMP-modification was not observed in the mutant peptide 150-DRHQEEARNAGDDDFAVSNDY-170 (Supplementary Figure 11).

Supplementary References

1 Lehmann, K. C. *et al.* Discovery of an essential nucleotidylating activity associated with a newly delineated conserved domain in the RNA polymerase-containing protein of all nidoviruses. *Nucleic Acids Res* **43**, 8416-8434, doi:10.1093/nar/gkv838 (2015).

2 Posthuma, C. C., Te Velthuis, A. J. W. & Snijder, E. J. Nidovirus RNA polymerases: Complex enzymes handling exceptional RNA genomes. *Virus Res* **234**, 58-73, doi:10.1016/j.virusres.2017.01.023 (2017).

3 McGinnis, S. & Madden, T. L. BLAST: at the core of a powerful and diverse set of sequence analysis tools. *Nucleic Acids Res* **32**, W20-25, doi:10.1093/nar/gkh435 (2004).

4 Wheeler, D. L. *et al.* Database resources of the National Center for Biotechnology Information: update. *Nucleic Acids Res* **32**, D35-40, doi:10.1093/nar/gkh073 (2004).

5 Madeira, F. *et al.* The EMBL-EBI search and sequence analysis tools APIs in 2019. *Nucleic Acids Res* **47**, W636-W641, doi:10.1093/nar/gkz268 (2019).

6 Peti, W. *et al.* Structural genomics of the severe acute respiratory syndrome coronavirus: nuclear magnetic resonance structure of the protein nsP7. *J Virol* **79**, 12905-12913, doi:10.1128/JVI.79.20.12905-12913.2005 (2005).

7 Gao, Y. *et al.* Structure of the RNA-dependent RNA polymerase from COVID-19 virus. *Science* **368**, 779-782, doi:10.1126/science.abb7498 (2020).

8 Manolaridis, I. *et al.* Structure and genetic analysis of the arterivirus nonstructural protein 7alpha. *J Virol* **85**, 7449-7453, doi:10.1128/JVI.00255-11 (2011).

9 van Aken, D., Zevenhoven-Dobbe, J., Gorbalenya, A. E. & Snijder, E. J. Proteolytic maturation of replicase polyprotein pp1a by the nsp4 main proteinase is essential for equine arteritis virus replication and includes internal cleavage of nsp7. *J Gen Virol* **87**, 3473-3482, doi:10.1099/vir.0.82269-0 (2006).

10 Zhang, M. *et al.* Mutagenesis analysis of porcine reproductive and respiratory syndrome virus nonstructural protein 7. *Virus Genes* **47**, 467-477, doi:10.1007/s11262-013-0957-4 (2013).

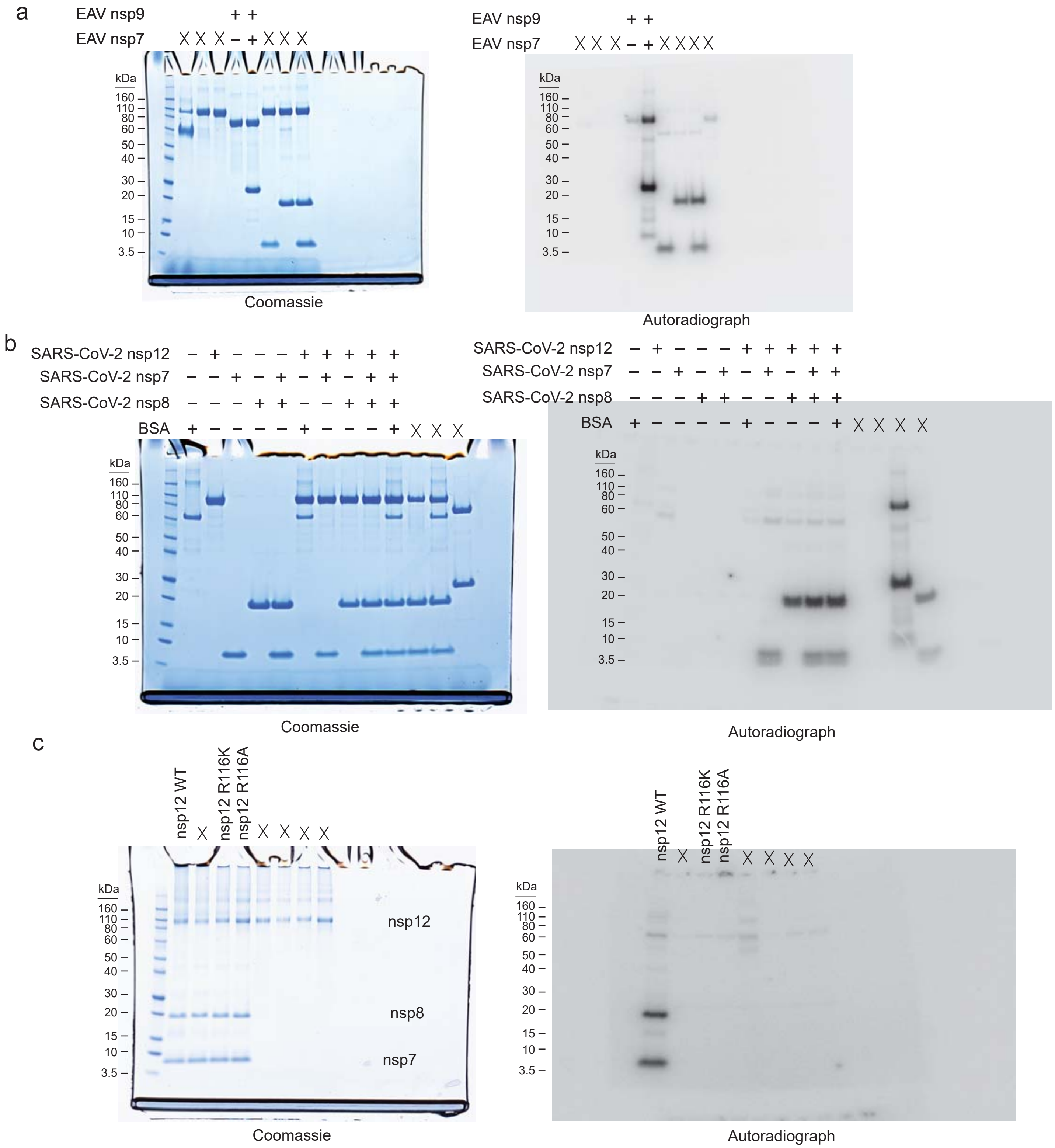

Supplementary Figure 1. Uncropped gels corresponding to Figure 1. Panels a, b, and c correspond to Coomassie stained and autoradiography gels in Figure 1a, 1b, and 1c. respectively. Note that two distinct gels were run for each experiment, one for Coomassie and one for autoradiography, and in panels a and b, extra samples were run on the autoradiography gel. “X” denotes lanes that are not depicted in Figure 1.

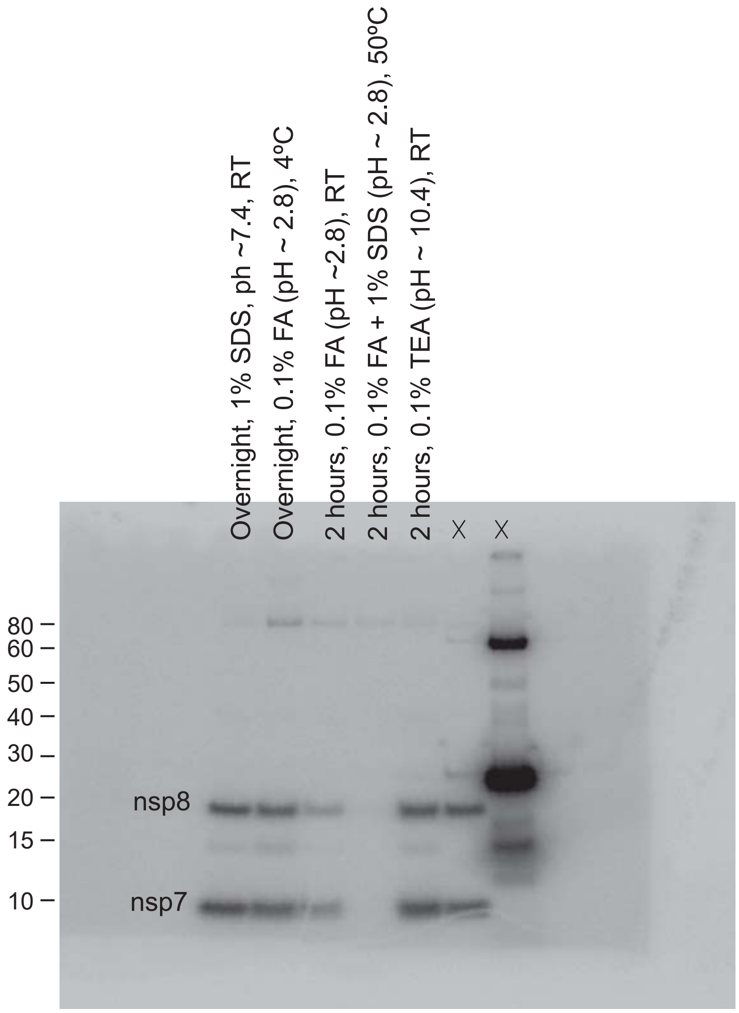

Supplementary Figure 2. Stability of GMP covalent attachment on SARS-CoV-2 nsp7 and nsp8. a) SARS-CoV-2 proteins nsp7 and nsp8, radiolabeled with ^{32}P-GTP, were incubated in the indicated conditions and ran on SDS-PAGE. Autoradiography was used to visualize radioactive proteins. Abbreviations: FA, formic acid; TEA, triethylamine; RT, room temperature. "X" denotes lanes that represents a different experiment performed on the same day. Uncropped gel image is shown.

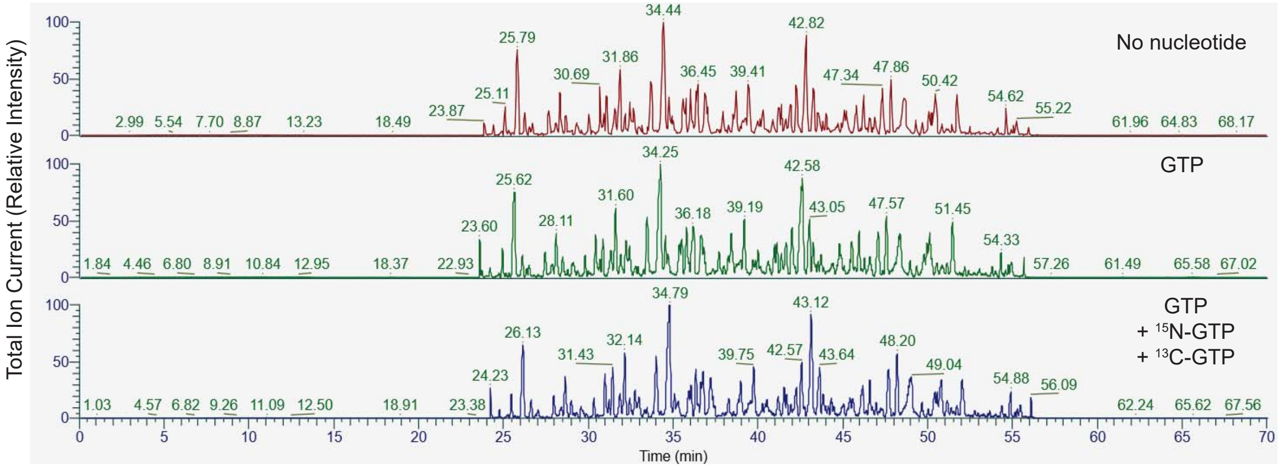

Supplementary Figure 3. Total Ion Chromatograms for SARS-CoV-2 nucleotidylated proteins. The total ion chromatograms for Figure 2a and 2b are shown that were subjected to nucleotidylation reactions, as described in the text, and digested with chymotrypsin. The overall peak profiles were similar and peptides eluted at similar retention times between 24 and 57 minutes.

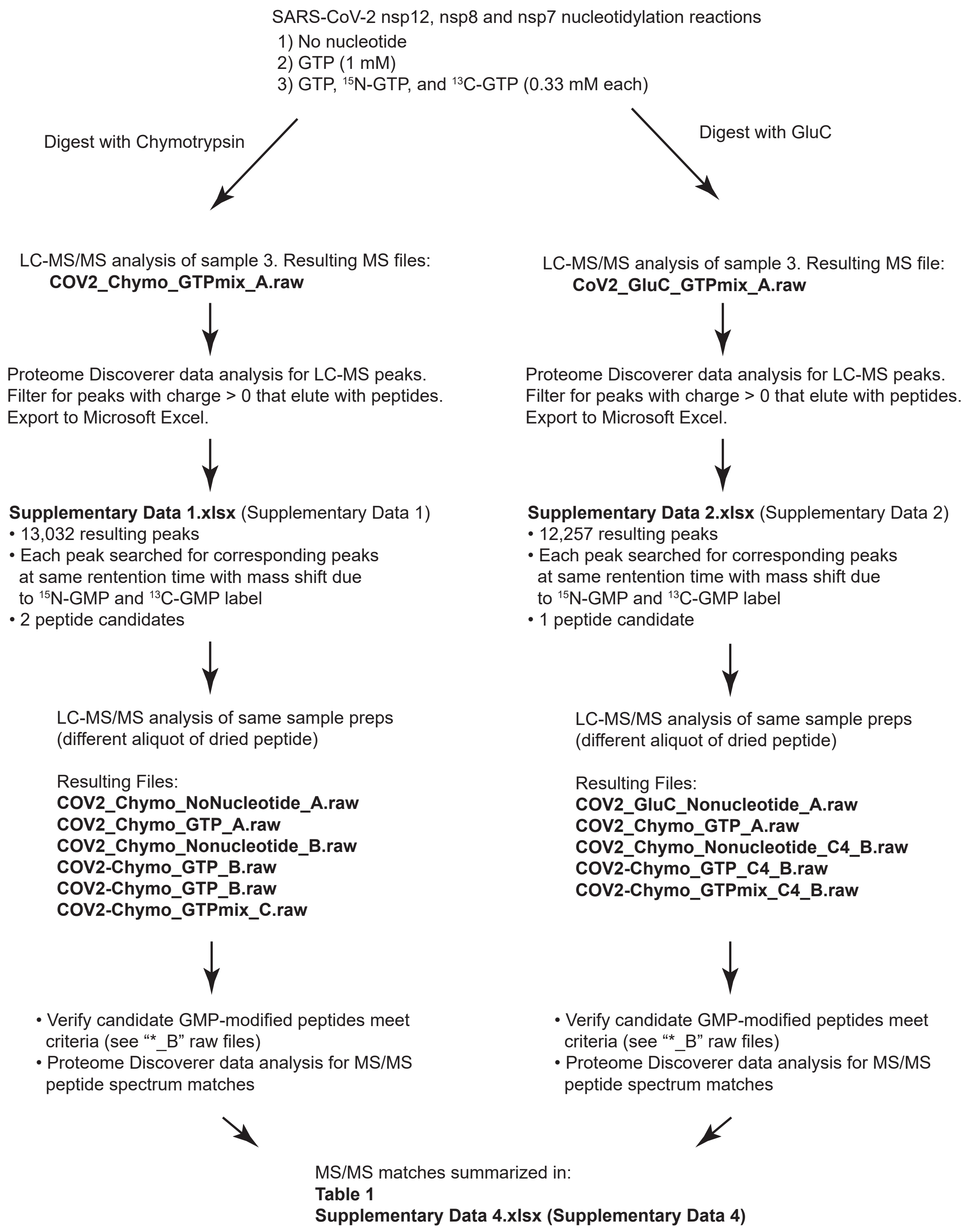

Supplementary Figure 4. Workflow for identification of SARS-CoV-2 GMP-labeled peptides. See files and tables indicated for raw data, analysis and results.

EAV nsp9 and nsp7 nucleotidylation reactions

1) No nucleotide
2) GTP (0.2 mM)
3) ^{15}N-GTP (0.2 mM)
4) ^{13}C-GTP (0.2 mM)

↓ Digest with Trypsin / LysC

LC-MS/MS analysis: 2 replicate injections per sample:

Resulting MS files:

EAV_Tryp_Nonucleotide_A.raw
EAV_Tryp_Nonucleotide_B.raw
EAV_Tryp_GTP_A.raw
EAV_Tryp_GTP_B.raw
EAV_Tryp_15NGTP_A.raw
EAV_Tryp_15NGTP_B.raw
EAV_Tryp_13CGTP_A.raw
EAV_Tryp_13CGTP_B.raw

Proteome Discoverer data analysis: 91,140 LC-MS peaks

Filtered for:

1) presence in both sample 2 replicates and absence in all other injections, or
2) presence in both sample 3 replicates and absence in all other injections, or
3) presence in both sample 4 replicates and absence in all other injections, or

Export to Microsoft Excel

↓

Supplementary Data 3.xlsx (Supplementary Data 3):
1491 LC-MS peaks

Each peak searched for corresponding peaks at same rentention time with mass shift due to ^{15}N-GMP and ^{13}C-GMP label

50 LC-MS peaks identified

Additional LC-MS/MS acquisitions:

EAV_Tryp_GTP_C.raw
EAV_Tryp_13CGTP_C.raw

↓

Proteome Discoverer data analysis for MS/MS peptide spectrum matches

Summarized in:
Table I
Supplemetary Data 4.xlsx
(Supplementary Data 4)

Supplementary Figure 5. Workflow for identification of EAV GMP-labeled peptides. See files and tables indicated for raw data, analysis, and results.

HCD fragmentation

SARS-Cov-2 nsp7
GMP-modified
GSKMSDVKC(+57)TSVVL
m/z 928.4072, charge = +2

	b - GMP⁺	b⁺	Sequence	y⁺	y - GMP⁺	
1		58.02874	G			14
2		145.06077	S	1798.78635	1453.73891	13
3	273.15573*	618.20317	K-Phosphoguanosine	1711.75432	1366.70688*	12
4	404.19622*	749.24365*	M	1238.61192*		11
5	491.22824*	836.27568	S	1107.57144*		10
6	606.25519*	951.30262*	D	1020.53941*		9
7	705.32360*	1050.37104	V	905.51247*		8
8	833.41856*	1178.46600*	K	806.44405*		7
9	993.44921*	1338.49665	C-Carbamidomethyl	678.34909*		6
10	1094.49689*	1439.54433	T	518.31844		5
11	1181.52892*	1526.57635	S	417.27076		4
12	1280.59733*	1625.64477	V	330.23873*		3
13	1379.66575*	1724.71318	V	231.17032*		2
14			L	132.10191		1

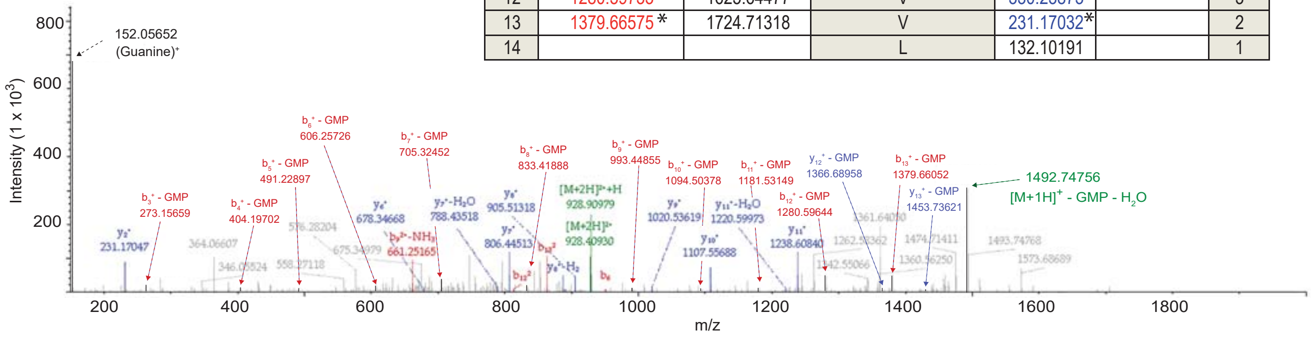

Supplementary Figure 6. HCD spectrum for GMP-modified SARS-CoV-2 nsp7 peptide 1-14. The top scoring HCD spectrum match for GMP-modified nsp7 peptide is shown for direct comparison to the EThcD spectrum (Figure 2d). Note that the modification site was assigned to K3 by Sequest HT for this spectrum. Guanine and fragments that lost the GMP modification were manually labeled after inspection. Red, blue and green coloring indicate b- fragment ions, y- fragment ions, and precursor ions, respectively, that were experimentally observed with a mass +/- 0.04 Da of the calculated, theoretical fragment ion mass. The inset table diplays these calculated masses for b- and y-series ions that could be generated following HCD fragmentation. Colored ion fragement masses, which are also denoted by asterisks, indicate ions that were observed experimentally.

	b^+	c^+	$c+H^+$	Seq.	y^+	y^{2+}	z^+	$z+H^+$	
1	58.02874	75.05529	76.06311	G					17
2	171.11280	188.13935	189.14718	L	2309.04588	1155.02658	2294.03498	2295.04281	16
3	268.16557	285.19212	286.19994	P	2195.96182	1098.48455*	2180.95092	2181.95875	15
4	741.30797	758.33452 *	759.34234*	K-Phosphoguanosine	2098.90906	1049.95817*	2083.89816	2084.90598	14
5	798.32943	815.35598 *	816.36380*	G	1625.76666	813.38697*	1610.75576*	1611.76358*	13
6	869.36654	886.39309 *	887.40092	A	1568.74519	784.87624*	1553.73430*	1554.74212	12
7	997.42512	1014.45167*	1015.45950*	Q	1497.70808	749.35768*	1482.69718	1483.70501	11
8	1110.50918	1127.53573*	1128.54356*	L	1369.64950	685.32839	1354.63860*	1355.64643*	10
9	1239.55178	1256.57833*	1257.58615*	E	1256.56544*	628.78636	1241.55454*	1242.56237*	9
10	1425.63109	1442.65764*	1443.66546*	W	1127.52285 *	564.26506*	1112.51195*	1113.51977 *	8
11	1540.65803	1557.68458*	1558.69241*	D	941.44353	471.22541	926.43263	927.44046	7
12	1696.75914	1713.78569	1714.79352	R	826.41659 *	413.71193*	811.40569*	812.41352	6
13	1833.81806	1850.84461	1851.85243*	H	670.31548*	335.66138	655.30458	656.31241	5
14	1961.87663	1978.90318	1979.91101*	Q	533.25657	267.13192	518.24567	519.25349	4
15	2090.91923	2107.94578	2108.95360	E	405.19799	203.10263	390.18709	391.19492	3
16	2219.96182	2236.98837	2237.99619	E	276.15540*	138.58134	261.14450	262.15232	2
17				K	147.11280	74.06004	132.10191	133.10973	1

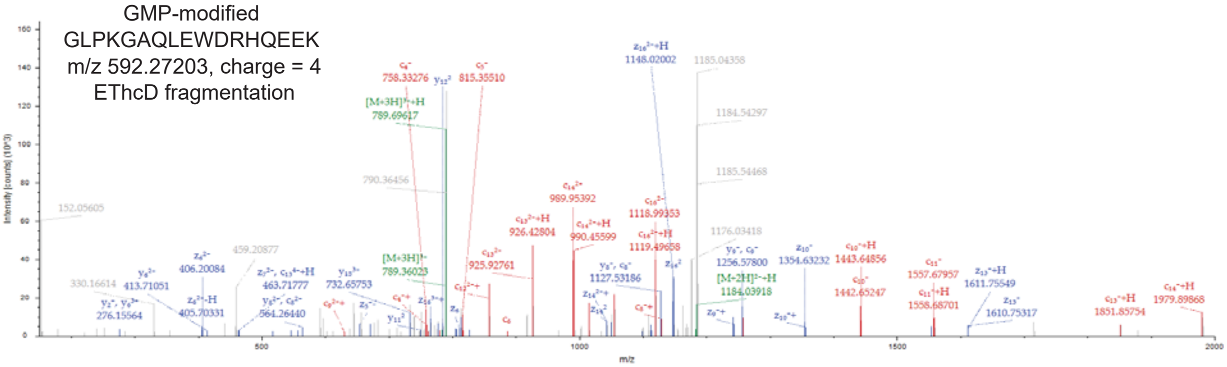

Supplementary Figure 7. EThcD spectrum for GMP-modified EAV nsp7 peptide 140-156. The top scoring EThcD spectrum match for the GMP-modified nsp7 peptide in Figure 3a is shown. Red, blue and green coloring indicate c- fragment ions, y- and z- fragment ions, and precursor ions, respectively, that were experimentally observed with a mass +/- 0.04 Da of the calculated, theoretical fragment ion mass. The inset table diplays these calculated masses for b-, c- , y- and z- series ions that could be generated following EThcD fragmentation. Colored ion fragements masses, which are also denoted by asterisks, indicate ions that were observed experimentally.

	b⁺	c⁺	c+H⁺	Seq.	y⁺	y²⁺	z⁺	z+H⁺	
1	58.02874	75.05529	76.06311	G					17
2	171.11280*	188.13935	189.14718	L	2319.07945	1160.04336*	2304.06855	2305.07637	16
3	268.16557	285.19212	286.19994	P	2205.99538	1103.50133*	2190.98449	2191.99231	15
4	751.34153	768.36808	769.37591	K-Phosphoguanosine (13C)	2108.94262	1054.97495	2093.93172	2094.93955	14
5	808.36299	825.38954 *	826.39737 *	G	1625.76666	813.38697 *	1610.75576 *	1611.76358 *	13
6	879.40011	896.42666 *	897.43448	A	1568.74519	784.87624 *	1553.73430 *	1554.74212	12
7	1007.45869	1024.48523 *	1025.49306 *	Q	1497.70808	749.35768	1482.69718	1483.70501	11
8	1120.54275	1137.56930*	1138.57712 *	L	1369.64950	685.32839	1354.63860 *	1355.64643*	10
9	1249.58534	1266.61189*	1267.61972 *	E	1256.56544	628.78636*	1241.55454 *	1242.56237 *	9
10	1435.66466	1452.69120 *	1453.69903*	W	1127.52285	564.26506*	1112.51195	1113.51977 *	8
11	1550.69160	1567.71815 *	1568.72597 *	D	941.44353 *	471.22541 *	926.43263 *	927.44046 *	7
12	1706.79271	1723.81926 *	1724.82708 *	R	826.41659 *	413.71193 *	811.40569 *	812.41352 *	6
13	1843.85162	1860.87817 *	1861.88600 *	H	670.31548 *	335.66138	655.30458*	656.31241	5
14	1971.91020	1988.93675 *	1989.94457 *	Q	533.25657	267.13192	518.24567	519.25349	4
15	2100.95279	2117.97934	2118.98717	E	405.19799	203.10263	390.18709	391.19492	3
16	2229.99538	2247.02193	2248.02976	E	276.15540*	138.58134	261.14450	262.15232	2
17				K	147.11280	74.06004	132.10191	133.10973	1

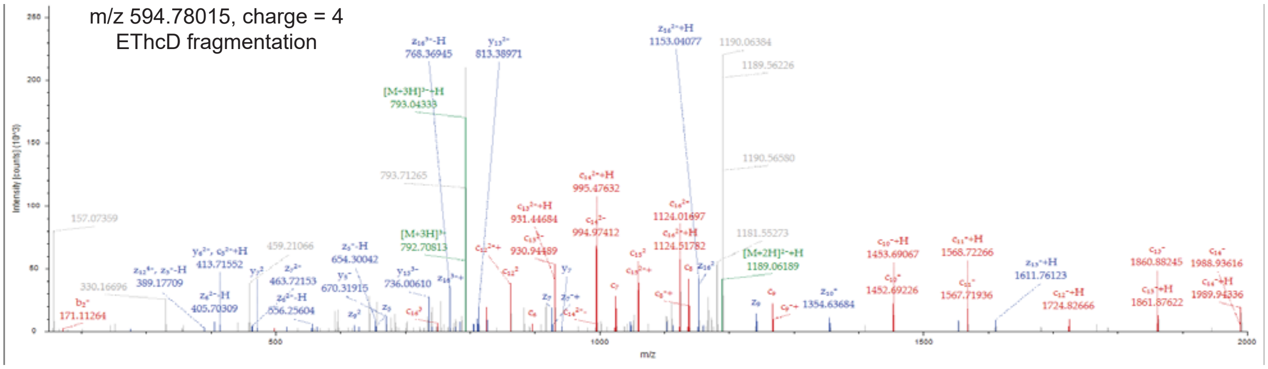

Supplementary Figure 8. EThcD spectrum for ^{13}C-GMP-modified EAV nsp7 peptide 140-156. The top scoring EThcD spectrum match for the ^{13}C-GMP-modified nsp7 peptide in Figure 3b is shown. Red, blue and green coloring indicate c- and b- fragment ions, y- and z- fragment ions, and precursor ions, respectively, that were experimentally observed with a mass +/- 0.04 Da of the calculated, theoretical fragment ion mass. The inset table diplays these calculated masses for b-, c- , y- and z- series ions that could be generated following EThcD fragmentation. Colored ion fragements masses, which are also denoted by asterisks, indicate ions that were observed experimentally.

	b$^+$	c$^+$	c+H$^+$	Seq.	y$^+$	z$^+$	z+H$^+$	z-H$^+$	
1	157.10839	174.13494	175.14276	R					17
2	271.15131	288.17786*	289.18569	N	2135.83643	2120.82553	2121.83336	2119.81771	16
3	342.18843	359.21498*	360.22280	A	2021.79350	2006.78260	2007.79043	2005.77478	15
4	399.20989	416.23644*	417.24427*	G	1950.75639	1935.74549*	1936.75332	1934.73767*	14
5	514.23684	531.26338*	532.27121*	D	1893.73493	1878.72403*	1879.73185*	1877.71620*	13
6	629.26378	646.29033*	647.29815*	D	1778.70798	1763.69708*	1764.70491*	1762.68926*	12
7	744.29072	761.31727*	762.32510*	D	1663.68104	1648.67014*	1649.67797	1647.66232*	11
8	891.35914	908.38568*	909.39351*	F	1548.65410	1533.64320*	1534.65102*	1532.63537*	10
9	962.39625	979.42280*	980.43062	A	1401.58568	1386.57478*	1387.58261*	1385.56696*	9
10	1061.46466	1078.49121*	1079.49904*	V	1330.54857	1315.53767	1316.54550	1314.52985*	8
11	1148.49669	1165.52324*	1166.53107*	S	1231.48016*	1216.46926*	1217.47708	1215.46143*	7
12	1262.53962	1279.56617*	1280.57399*	N	1144.44813*	1129.43723*	1130.44505	1128.42940*	6
13	1377.56656	1394.59311*	1395.60094*	D	1030.40520	1015.39430*	1016.40213	1014.38648*	5
14	1540.62989	1557.65644*	1558.66426	Y	915.37826	900.36736*	901.37518	899.35953*	4
15	1639.69830	1656.72485*	1657.73268*	V	752.31493	737.30403	738.31186	736.29621*	3
16	2117.82587	2134.85242	2135.86024	K-phosphoguanosine (15N)	653.24652*	638.23562	639.24344	637.22779*	2
17				R	175.11895*	160.10805	161.11588	159.10023*	1

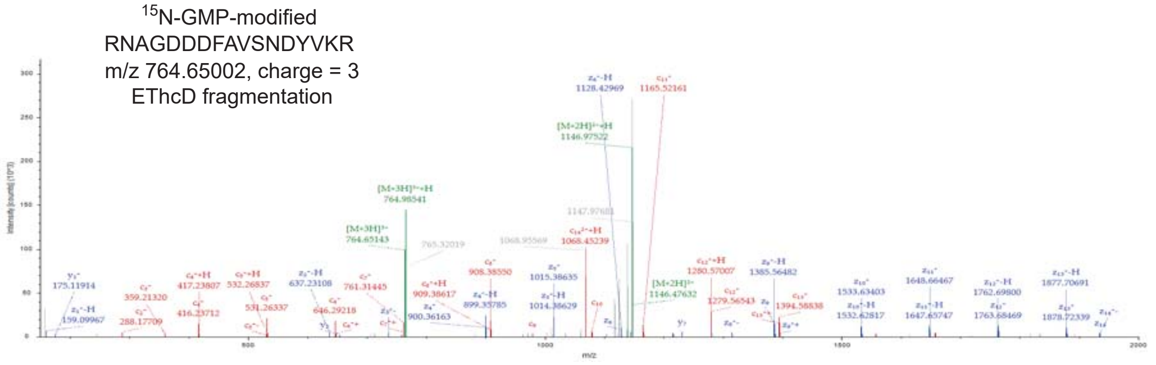

Supplementary Figure 9. EThcD spectrum for ^{15}N-GMP-modified EAV nsp7 peptide 157-173. The top scoring EThcD spectrum match for the ^{15}N-GMP-modified nsp7 peptide in Figure 3c is shown. Red, blue and green coloring indicate c- fragment ions, y- and z- fragment ions, and precursor ions, respectively, that were experimentally observed with a mass +/- 0.04 Da of the calculated, theoretical fragment ion mass. The inset table diplays these calculated masses for b-, c- , y- and z- series ions that could be generated following EThcD fragmentation. Colored ion fragements masses, which are also denoted by asterisks, indicate ions that were observed experimentally.

	b^+	b^{2+}	Sequence	y^+	y^+ - GMP	
1	157.10839 *	79.05783	R			17
2	271.15131 *	136.07930	N	2135.83643	1785.80383	16
3	342.18843	171.59785	A	2021.79350	1671.76090	15
4	399.20989 *	200.10858	G	1950.75639	1600.72379	14
5	514.23684 *	257.62206	D	1893.73493	1543.70233	13
6	629.26378 *	315.13553	D	1778.70798	1428.67538 *	12
7	744.29072 *	372.64900	D	1663.68104	1313.64844 *	11
8	891.35914 *	446.18321	F	1548.65410	1198.62150 *	10
9	962.39625	481.70176 *	A	1401.58568	1051.55308	9
10	1061.46466 *	531.23597	V	1330.54857	980.51597 *	8
11	1148.49669 *	574.75198	S	1231.48016	881.44756 *	7
12	1262.53962 *	631.77345	N	1144.44813	794.41553 *	6
13	1377.56656 *	689.28692	D	1030.40520	680.37260 *	5
14	1540.62989 *	770.81858	Y	915.37826	565.34566 *	4
15	1639.69830 *	820.35279	V	752.31493	402.28233 *	3
16	1767.79327 *	884.40027	K	653.24652	303.21392 *	2
17			R-Phosphoguanosine (N15)	525.15155	175.11895 *	1

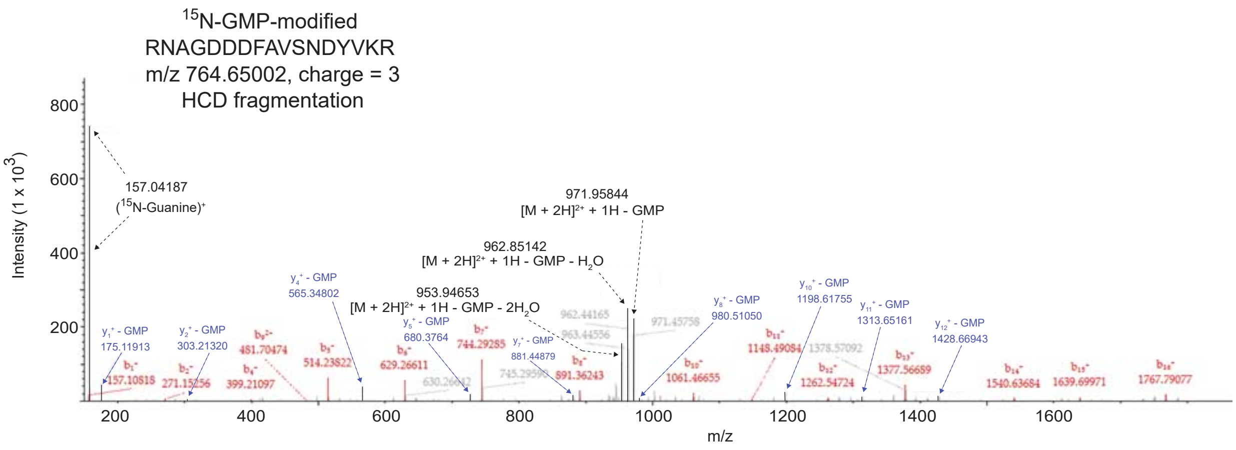

Supplementary Figure 10. HCD spectrum for ^{15}N-GMP-modified EAV nsp7 peptide 157-173 with GMP-modication loss labeling. The top scoring HCD spectrum match for the ^{15}N-GMP-modified nsp7 peptide in Figure 3c is relabeled with fragment ions that have lost the GMP modification. Red and blue coloring indicate b- fragment ions and y-fragment ions that were experimentally observed with a mass +/- 0.04 Da of the calculated, theoretical fragment ion mass. The inset table diplays these calculated masses for b- and y-series ions that could be generated following HCD fragmentation. Colored ion fragements masses, which are also denoted by asterisks, indicate ions that were observed experimentally.

a

EAV nsp7 WT →

EAV nsp7 WT, 1-SLTATLAALTDDDFQFLSDVLDCR-24 (trypsin)			
charge state	m/z	GMP	total area
2	1344.147	No	1.67E+08
*3	896.434		
4	672.578		
2	1516.671	Yes	1.21E+07 (6.7%)
*3	1011.450		
4	758.839		

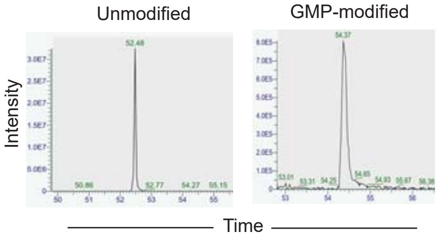

EAV nsp7 T3A →

EAV nsp7 T3A, 1-SLAATLAALTDDDFQFLSDVLDCR-24 (trypsin)			
charge state	m/z	GMP	total area
2	1329.142	No	6.03E+09
*3	886.43064		
4	665.07493		
2	1501.6657	Yes	2.09E+08 (3.3%)
*3	1001.4464		
4	751.33678		

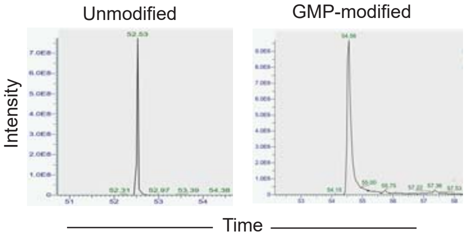

b

EAV nsp7 WT →

EAV nsp7 WT, 140-GLPKGAQLEWDR-151 (trypsin)			
charge state	m/z	GMP	total area
2	685.365	No	1.63E+06
*3	457.246		
4	343.1864		
2	857.8887	Yes	2.83E+06 (63.5%)
*3	572.2618		
4	429.4483		

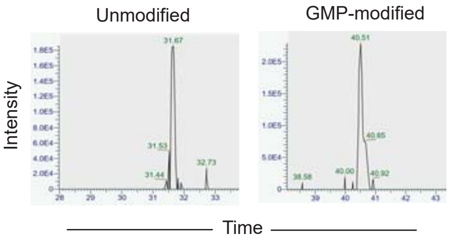

EAV nsp7 K143A →

EAV nsp7 K143A, 140-GLPAGAQLEWDR-150 (trypsin)			
charge state	m/z	GMP	total area
1	1312.664	No	2.77E+08
*2	656.8361		
3	438.2267		
1	1657.712	Yes	Not Found
*2	829.3598		
3	553.2425		

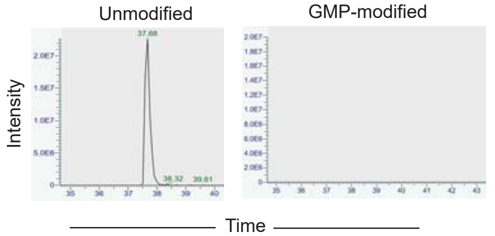

c

EAV nsp7 WT →

EAV nsp7 WT, 150-DRHQEEKRNAGDDDFAVSNDY-170 (chymotrypsin)			
charge state	m/z	GMP	total area
2	1241.037	No	7.48E+09
3	827.6939		
*4	621.0224		
2	1413.561	Yes	9.10E+07 (1.2%)
3	942.7097		
*4	707.2842		

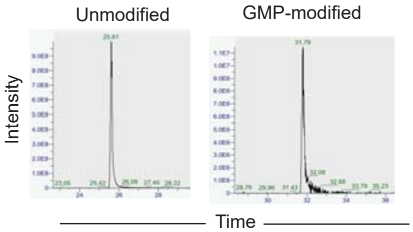

EAV nsp7 K156A →

EAV nsp7 K156A, 150-DRHQEEARNAGDDDFAVSNDY-170 (chymotrypsin)			
charge state	m/z	GMP	total area
2	1212.508	No	2.96E+09
3	808.6746		
*4	606.7579		
2	1385.032	Yes	Not Found
3	923.6904		
*4	693.0198		

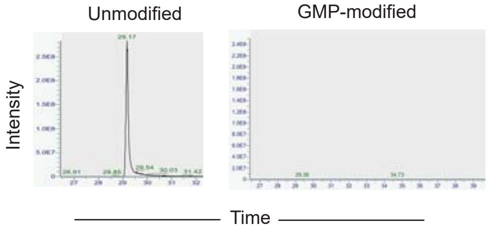

d

EAV nsp7 WT →

EAV nsp7 WT, 158-NAGDDDFAVSNDYVKR-173 (trypsin)			
charge state	m/z	GMP	total area
2	893.4058	No	2.02E+08
*3	595.940		
4	447.2068		
2	1065.93	Yes	1.46E+6 (0.7%)
*3	710.9556		
4	533.4687		

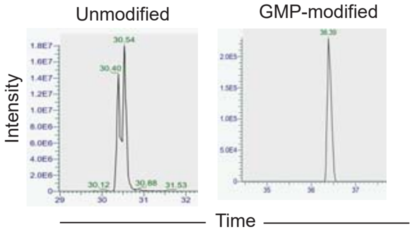

EAV nsp7 K172A →

EAV nsp7 K172A, 158-NAGDDDFAVSNDYVAR-173 (trypsin)			
charge state	m/z	GMP	total area
*2	864.8769	No	3.13E+09
3	576.9205		
4	432.9423		
*2	1037.401	Yes	Not Found
3	691.9363		
4	519.2042		

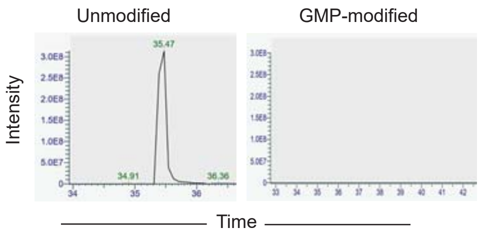

e

EAV nsp9 WT →

EAV nsp9 WT, 369-SNLQTATMATCKR-381 (trypsin)			
charge state	m/z	GMP	total area
2	741.3638	No	1.32E+09
3	494.5785		
*4	371.1858		
2	913.8875	Yes	9.77E+06 (0.7%)
3	609.5943		
*4	457.4477		

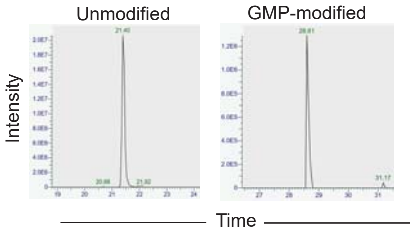

EAV nsp9 K380A →

EAV nsp9 K380A, 369-SNLQTATMATCAR-381 (trypsin)			
charge state	m/z	GMP	total area
*2	712.8349	No	4.38E+09
3	475.5592		
4	356.9214		
*2	885.3586	Yes	Not Found
3	590.575		
4	443.1832		

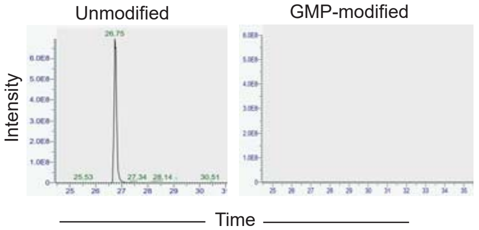

Supplementary Figure 11. LC-MS data for WT and mutant EAV nsp proteins. GMP-labeled EAV WT protein digests were compared to similar samples prepared from those containing (a) EAV nsp7 T3A, (b) EAV nsp7 K143A, (c) EAV nsp7 K156A, (d) EAV nsp7 K172A, or (e) EAV nsp9 K380A. For each mutant, one tryptic or chymotryptic peptide containing the mutation is shown. The m/z values for these peptides were calculated for charge states 1-5 (only three charges states are shown). The LC-MS peak of the most abundant m/z peak (indicated by an asterisk) was extracted from the raw data for both the unmodified and GMP-modified peptides as labeled. The only EAV nsp mutant that was still modified by GMP was EAV nsp7 T3A. Total area of all m/z peaks were calculated and the GMP-modified percentage was denoted in brackets. For the EAV nsp7 K156 GMP-modification site, a chymotryptic peptide containing the site was analyzed because the originally tryptic peptide was not observed in samples for unknown reasons. The percentage of GMP-modified peptide for K143 is likely overestimated because tryptic cleavage at K143 could occur in the absence of the GMP-modification, as opposed to the 140-151 peptide where the GMP modification causes a missed cleavage event.

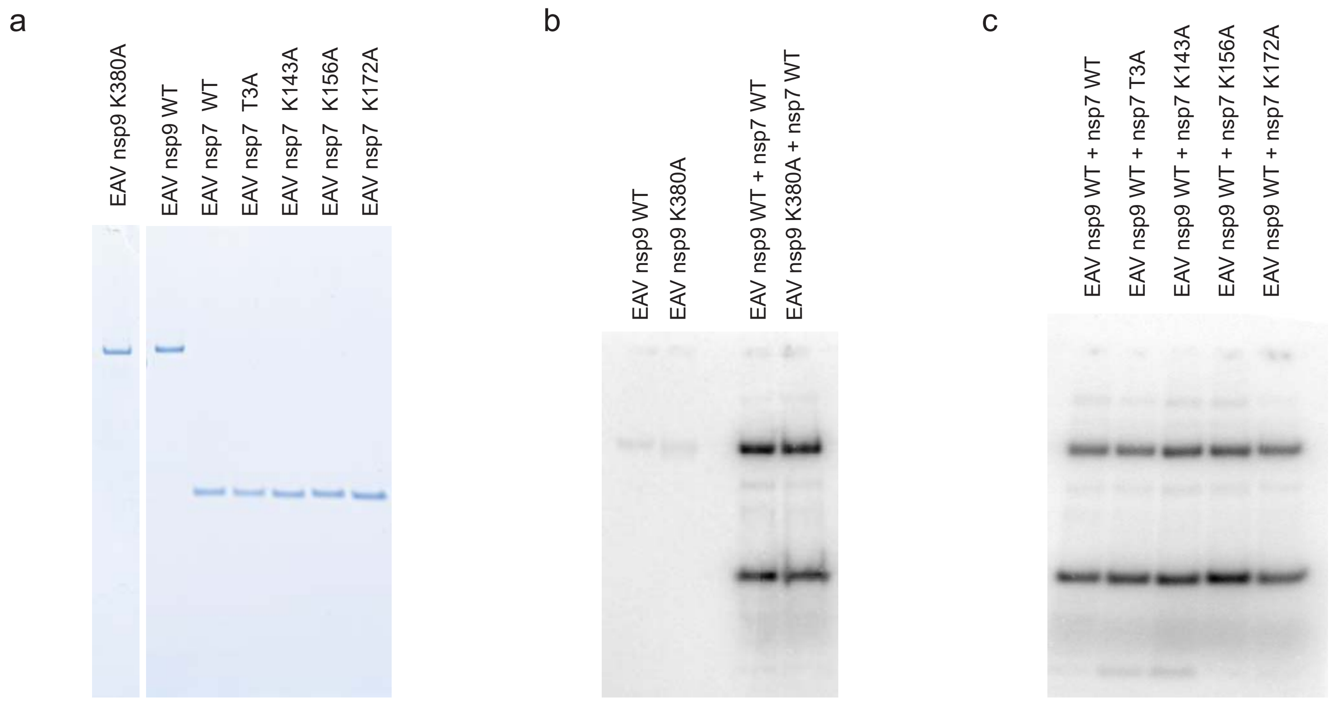

Supplementary Figure 12. EAV nsp7 and nsp9 mutant proteins. a) Purified EAV nsp7 and nsp9 WT proteins were compared to equal quantities of EAV nsp9 K380A, EAV nsp7 T3A, EAV nsp7 K143A, EAV nsp7 K156A, and EAV nsp7 K172A. Proteins were analyzed by SDS-PAGE and stained with Coomassie. b) GMP radiolabeling of EAV nsp9 WT compared to EAV nsp9 K380A in the absences and presence of EAV nsp7 WT. c) GMP radiolabeling of EAV nsp7 WT and mutant proteins in the presence of EAV nsp9. Uncropped gels are shown in Supplementary Figure 13.

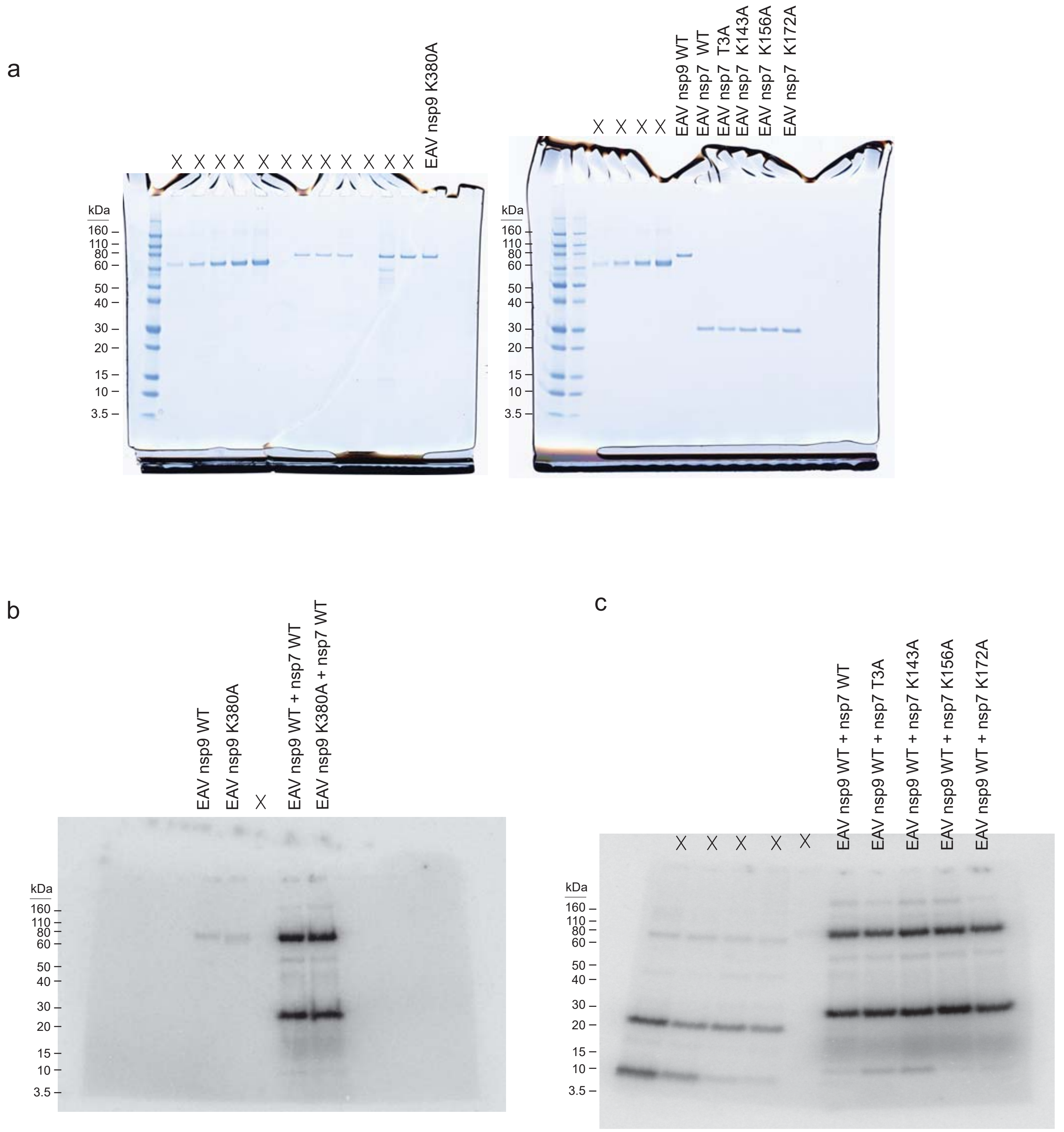

Supplementary Figure 13. Uncropped gels for Supplementary Figure 12. Panels a, b, and c correspond to Coomassie stained and autoradiography gels in Figure 12a, 12b, and 12c. respectively.

a

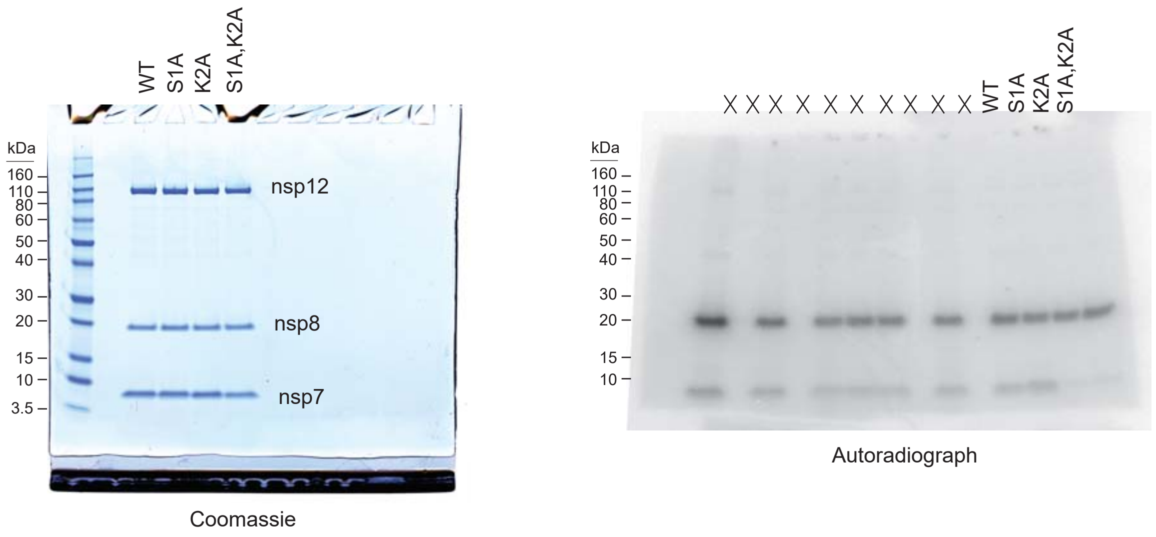

Supplementary Figure 14. Uncropped gels of Figure 4.
Panel a corresponds to Coomassie stained and autoradiography gel in Figure 4a.

a

SARS-CoV-2 nsp7 WT, 1-G**S**KMSDVKCTSVVL-14 (chymotrypsin)			
charge state	m/z	GMP	total area
1	1510.76	No	5.02E+09
2	755.8841		
*3	504.2587		
1	1855.808	Yes	1.30E+07 (0.26%)
*2	928.4078		
3	619.2745		

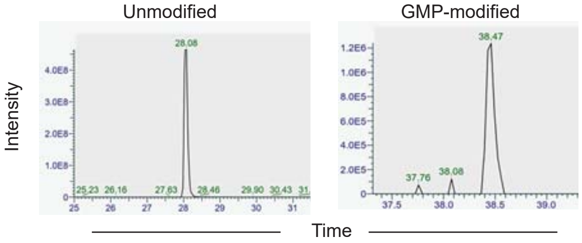

b

SARS-CoV-2 nsp7 S2A, 1-G**A**KMSDVKCTSVVL-14 (chymotrypsin)			
charge state	m/z	GMP	total area
1	1494.765	No	1.90E+10
*2	747.8866		
3	498.927		
1	1839.813	Yes	4.64E+07 (0.24%)
*2	920.4103		
3	613.9428		

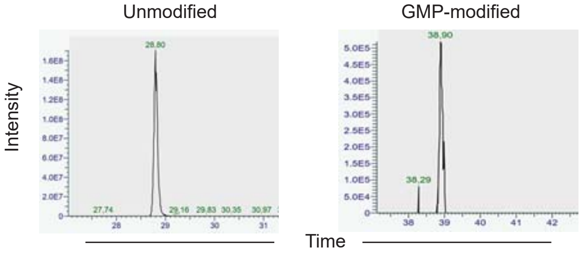

c

SARS-CoV-2 nsp7 K3A, 1-GS**A**MSDVKCTSVVL-14 (chymotrypsin)			
charge state	m/z	GMP	total area
1	1453.702	No	1.90E+10
*2	727.3551		
3	485.2394		
1	1798.75	Yes	5.38E+06 (0.028%)
*2	899.8788		
3	600.2552		

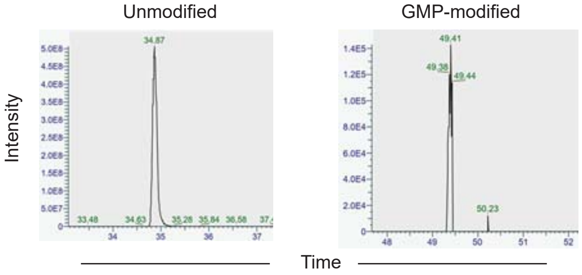

d

SARS-CoV-2 nsp7 S2AK3A, 1-G**AA**MSDVKCTSVVL-14 (chymotrypsin)			
charge state	m/z	GMP	total area
1	1437.708	No	2.70E+10
*2	719.3577		
3	479.9077		
1	1782.755	Yes	7.69E+06 (0.028%)
*2	891.8814		
3	594.9235		

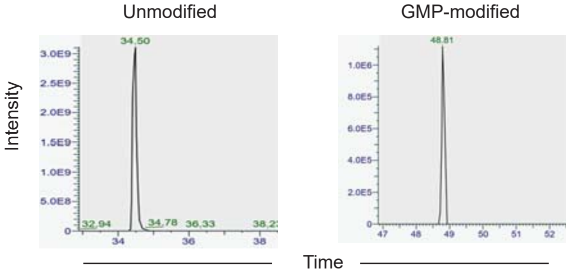

Supplementary Figure 15. LC-MS data for WT and mutant SARS-CoV-2 nsp7 proteins. GMP-labeled SARS-CoV-2 WT protein digests (a) were compared to similar samples prepared from those containing (b) SARS-CoV-2 nsp7 S2A, (c) SARS-CoV-2 nsp7 K3A, and (d) SARS-CoV-2 nsp7 S2AK3A. For each mutant, the chymotryptic peptide 1-14 containing the mutation is shown. The m/z values for these peptides were calculated for charge states 1-5 (only three charges states are shown). The LC-MS peak of the most abundant m/z peak (indicated by an asterisk) was extracted from the raw data for both the unmodified and GMP-modified peptides as labeled. Total area of all m/z peaks were calculated, and the GMP-modified percentage was denoted in brackets.

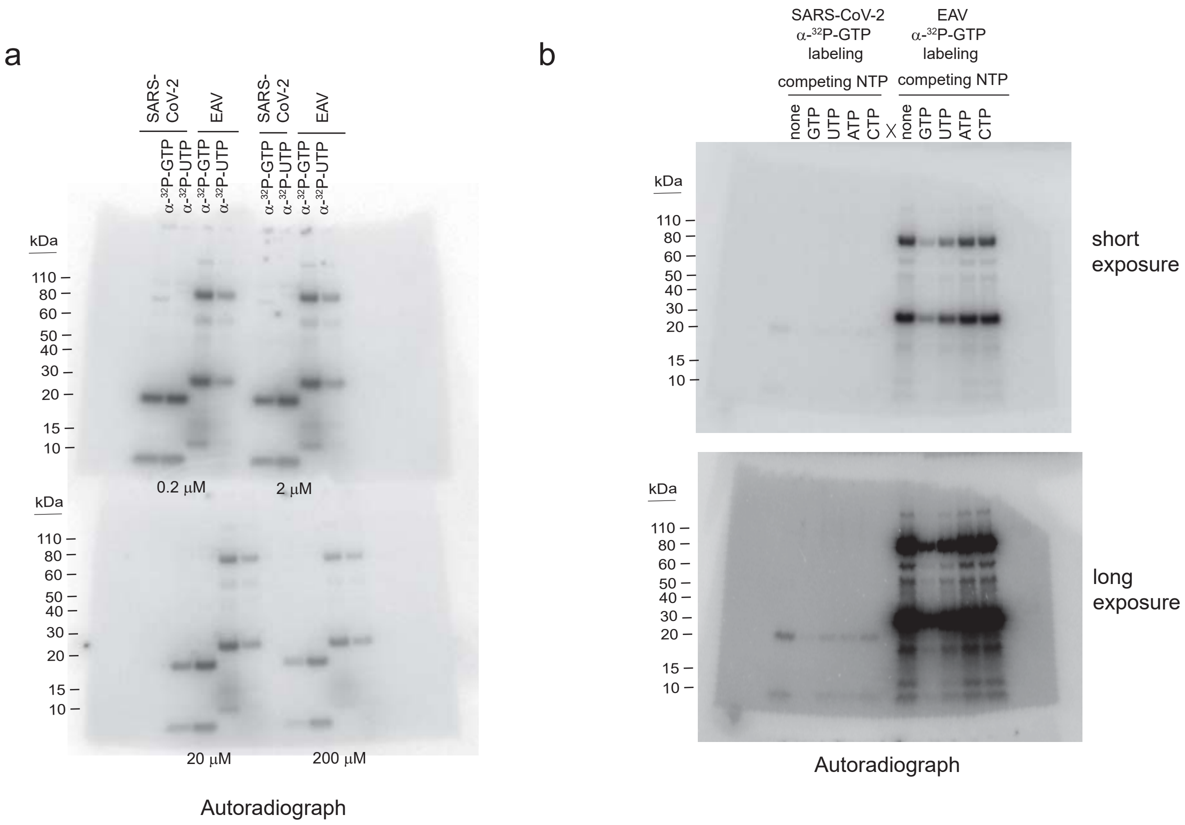

Supplementary Figure 16. Uncropped gels of Figure 5. Panels a and b correspond to autoradiography gels in Figure 5a and 5b, respectively.

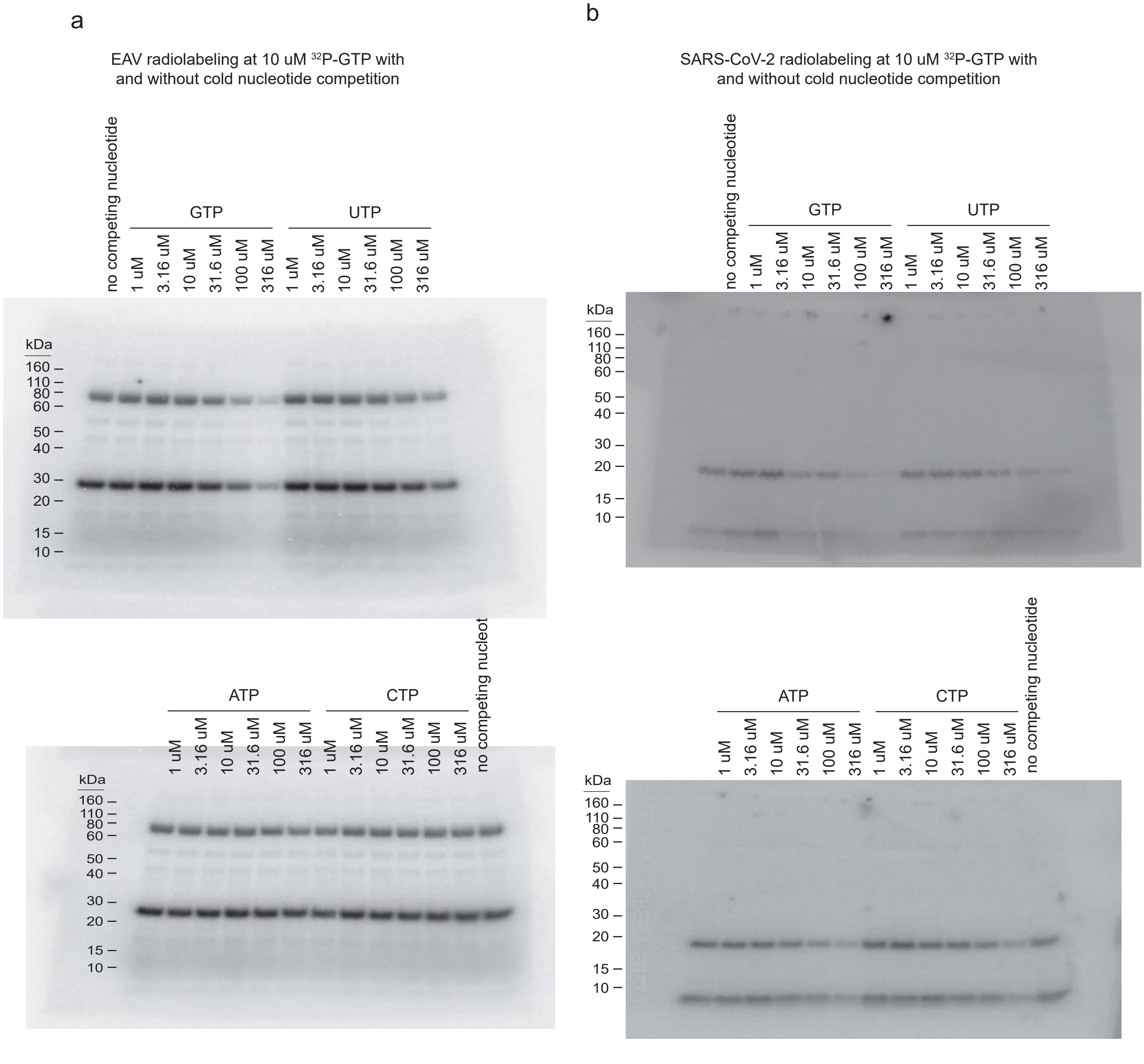

Supplemental Figure 17. Competition of GTP radiolabeling with cold GTP, UTP, ATP or CTP. Radiolabeling of EAV (a) or SARS-CoV-2 (b) proteins with α-^{32}P-GTP was competed against 1 μM to 316 μM cold nucleotide (GTP, UTP, ATP or CTP). Radioactive GTP concentrations (10 μM) were in excess of protein concentrations (0.6 to 2.7 μM). Labeled proteins were analyzed with SDS-PAGE followed by autoradiography. Uncropped gels are shown.

```
                                   ↓
ref|YP_005352880.1|       AISTVQNRVLDAKATAVIVANLLEKAGVTNKHVVCKKIVKLHNDTLKAATYEEAETALVK    3049
ref|YP_002308505.1|       AISTVQNKILDAKATAVVVANLLEKAGVTNKHAICKKIVKLHNETLKATTYQDAETSLVK    3187
ref|YP_002308478.1|       AVSTVQNKLLDAKAAAVVVANLLDKAGVTNKHAVCKKIVKLHNDTLKASTYEEAEMSLVK    3103
ref|YP_005352845.1|       AVSTVQNKILDAKATAVVVANLLEKAGVTNKHAVCKKIVKFHNDTLKATSYEEAEVALVK    3156
ref|YP_009019180.1|       KIASVQSKLTDMKCTNVVLLGLLSKMHVEANSKEWNYCVTLHNEINLSDDPDVVLNKLLA    3529
gb|ACT10947.1|            KISTVQSKLTEMKCTNVVLLGLLSKMHVESNSKEWNYCVGLHNEINLCDDPEVVLEKLLA    3540
ref|YP_001718603.1|       KISTVQSKLTDIKCTNVVLMGCLSSMNIEANSKKWSYCVDLHNKINLCDDAEKAMEYLLA    3782
ref|YP_001718610.1|       KISTVQSKLTEIKCTNVVLMGVLTSMNIEANSKDWAFCVDLHNKINLSTDAEKAMEYLLA    3745
ref|YP_008439200.1|       KISTVQSKLTDVKCTNVVLMGCLSSMNIQANSAEWNYCVDLHNKINLCNDLERAQEYLLA    3675
gb|AFU92112.1|            KVSSVQSKLTDIKCTNVVLLGCLSSMNIAANTKEWSYCVDLHNKINLCNDPEVAQEMLLA    3629
gb|AGK89913.1|            KISSVQSKLTDIKCSNVVLLGCLSSMNVSANSTEWAYCVDLHNKINLCNDPEKAQEMLLA    3633
ref|YP_001351683.1|       KISSVQSKLTDLKCTNVVLLGCLSNMNIAANSREWAYCVDLHNKINLCNDAEAAQEMLLA    3644
ref|YP_001552234.1|       KVSTVQSKLTDLKCANVVLLGCLTNMNIAANSREWAYCVNLHNEINLTSDPEEALEKLLA    3575
ref|YP_003766.2|          KISTVQSKLTDLKCTNVVLLGCLSSMNIAANSSEWAYCVDLHNKINLCDDPEKAQSMLLA    3575
gb|AFI49429.1|            KVSTVQSKLTDLKCTNVVLMGILSNMNIASNSKEWAYCVETHNKINLCDNPETAQELLLA    3604
gb|AAF19383.1|            EVSQIQSRLTDVKCVNVVLLNCLQHLHIASSSKLWQYCSTLHNEILATSDLSVAFDKLAQ    3923
gb|AAX76519.1|            EVSQIQSKLTDVKCANVVLLNCLQHLHVASNSRLWQYCSILHNEILSTSDLSVAFDKLAQ    3948
gb|AFE48810.1|            EVSQIQSKLTDVKCANVVLLNCLQHLHVASNSKLWQYCSTLHNEILATSDLSTAFEKLAQ    3950
gb|AGK85497.1|            PISTVQSKLSDVECTTVVLMQLLTKLNVEANSKMHAYLVELHNKILASDDVTECMDNLLG    3432
gb|AHB63480.1|            TVSTVQSKLTDVKCATVVLMQLLTKLNVEANSRLHKHLVQTHNDILAESDPAICIEKLTG    3419
gb|ABG47051.1|            KVATVQSKLTDLKCTSVVLLTVLQQLHLESNSKAWSYCVKLHNEILAAVDPTEAFERFVC    3950
ref|YP_009513008.1|       KIASVQSKLTDLKCTSVVLLSVLQQLHLEANSKAWSHCVKLHNDILSTSDPSEAFEKFVA    3962
ref|YP_009047202.1|       KVAAMQSKLTDLKCTSVVLLSVLQQLHLEANSRAWAFCVKCHNDILAATDPSEAFEKFVS    3899
gb|ABN10874.1|            KIASVQSKLTDLKCTSVVLLSVLQQLHLEANSKAWAHCVKLHNDILAATDPTEAFDNFVC    3990
gb|ADM33581.1|            KVSTVQSNMTDLKCTSVVLLSVLQQLRVEASSKLWSLCVKLHNEILASSSPSEAFEAFVS    3728
gb|AAP50483.1|            KVATVQSKMSDVKCTSVVLLSVLQQLRVESSSKLWAQCVQLHNDILLAKDTTEAFEKMVS    3890
lcl|SARS-CoV-2 nsp7       ------SKMSDVKCTSVVLLSVLQQLRVESSSKLWAQCVQLHNDILLAKDTTEAFEKMVS    54
ref|YP_009724389.1|       KVATVQSKMSDVKCTSVVLLSVLQQLRVESSSKLWAQCVQLHNDILLAKDTTEAFEKMVS    3913
                                ..: : :.  *::   *    :  .         **.             :
                                ↑
                                lys-2

ref|YP_005352880.1|       LLAHIIEFLPSEQVDEYLSMVNKPRVLDEYMDVLVQNKFVLQAVVDANIHMDSYRIFKEA    3109
ref|YP_002308505.1|       LLAHIVEFLPTDQVDAYLADEEEAQHVNNYLDNLLENTTVVQAVADANINLDSYRIYKEA    3247
ref|YP_002308478.1|       LLTHIIEFLPTDQVDAYLADTVKVQALNTYFDHLLENKLVLQAVVDANINLDSYRVYKEA    3163
ref|YP_005352845.1|       LLAHIIEFLPTDQVDAYLADAAKAQHVNTYLDNLLENKVVVQAVADININLDSYRIYKEA    3216
ref|YP_009019180.1|       LLAFFLSKHNNCD-------------LSELIDSYFDNASILQSVASAYASLPSWVAYEQA    3576
gb|ACT10947.1|            LIAFFLSKHNTCD-------------LSDLIDSYFENTTILQSVASAYAALPSWIAYEKA    3587
ref|YP_001718603.1|       LVTFFISEHADFN-------------VSELVDSYFGDNSILQSVASTFVNMPSFIAYESA    3829
ref|YP_001718610.1|       LLTFFLSRQKDFN-------------CTELLDSYFADSSILQSVASTFVNMPSFIAYETA    3792
ref|YP_008439200.1|       LLAFFLSKNSAFG-------------LDDLLDSYFDNNTVLQAVATTYANMPSYIMYENA    3722
gb|AFU92112.1|            LLAFFLSKHKDFG-------------FDDLLDSYFADNSILQSVASTFVNMPSFIAYENA    3676
gb|AGK89913.1|            LLAFFLSKNSAFG-------------LDDLLESYFNDNSMLQSVASTYVGLPSYVIYENA    3680
ref|YP_001351683.1|       LLAFFLSKNSAFG-------------VDELLDSYFNDSSVLQSVAATYVNLPSYLAYETA    3691
ref|YP_001552234.1|       LVAFFLSKQQNFG-------------VDDLIDSFFENRNVLQSVASAFANMPSFIAYEKA    3622
ref|YP_003766.2|          LLAFFLSKHSDFG-------------LDGLIDSYFDNSSTLQSVASSFVSMPSYIAYENA    3622
gb|AFI49429.1|            LLAFFLSKHSDFG-------------LGDLVDSYFENDSILQSVASSFVGMPSFVAYETA    3651
gb|AAF19383.1|            LLVVLFANPAAVD-------SKCLASIEEVSDDYVRDSTVLQALQSEFVNMASFVEYELA    3976
gb|AAX76519.1|            LLIVLFANPAAVD-------TKCLASIDEVSDDYVQDSTVLQALQSEFVNMASFVEYEVA    4001
gb|AFE48810.1|            LLIVLFANPAAVD-------SKCLSSIEEVCDDYAKDNTVLQALQSEFVNMASFVEYEVA    4003
gb|AGK85497.1|            MLVTLFCIDSTID-------------LSEYCDDILKRSTVLQSVTQEFSHIPSYAEYERA    3479
gb|AHB63480.1|            MLMTLLSIDSTLD-------------VKALCDELLAKESVLQAVTDEFCALPSYVDYEKA    3466
gb|ABG47051.1|            LFATLMSFSANVD-------------LDALANDLFENSSVLQATLTEFSHLATYAELETA    3997
ref|YP_009513008.1|       LLATLMSFSGSVD-------------LEALASELLDNISVLQSTLTEFSHLASYAELETA    4009
ref|YP_009047202.1|       LFATLMTFSGNVD-------------LDALASDIFDTPSVLQATLSEFSHLATFAELEAA    3946
gb|ABN10874.1|            LFATLMSFSANVD-------------LEALASDLLDHPSVLQATLSEFSHLASYAELEAA    4037
gb|ADM33581.1|            LLSVLLSLPGAIN-------------LDELCNSILENNTVLQAVASEFSNLSSYVDYENA    3775
gb|AAP50483.1|            LLSVLLSMQGAVD-------------INRLCEEMLDNRATLQAIASEFSSLPSYAAYATA    3937
lcl|SARS-CoV-2 nsp7       LLSVLLSMQGAVD-------------INKLCEEMLDNRATLQ------------------    83
ref|YP_009724389.1|       LLSVLLSMQGAVD-------------INKLCEEMLDNRATLQAIASEFSSLPSYAAFATA    3960
                          :.  :.                         .        :*
```

Supplementary Figure 18. Multiple sequence alignment of SARS-CoV-2 nsp7. Within nidoviruses, SARS-CoV-2 nsp7 aligns only with other coronavirus polyproteins, where lys-2 is conserved, except in the common moorhen coronavirus HKU21 (YP_005352880.1), murine hepatitis virus strain 2 (AAF19383.1) and Bat coronavirus HKU9-10-2 (ADM33581.1). In these strains, an arginine or asparagine are found instead of a lysine. The SARS-CoV-2 nsp7 and polyprotein 1ab sequences (YP_009724389) are both included as separate entries in the alignment. "*" indicates complete conservation, ":" indicates strong conservation, and "." indicates weaker conservation per the Clustal Omega algorithm.

```
YP_001661452.1        SLSDNTSFITAQIEHIKHHFSVWLFEVIPKISIQPVDKALRSIFIGPAFMNDVYRCFNTA       4705
YP_009666324.1        SLSDYTSFIRSQIEHIKHHFSIWLFEVIPKISIQPVDKALRSIFIGPAFMNDVYRCFNTA       4717
YP_008798230.1        RA-LGPENIMKLFEYAQKAPLPFCTKIITKFALSAKARA-RTVSSCSFIASTIFRFAHKP       4912
YP_337905.2           RA-LGPENITKLFEYAQKAPLPFCTKIITKFALSAKARA-RTVSSCSFIASTIFRFAHKP       4940
ABI97394.1            NA-LPEDFSPRLLDTASKTVMPFSTNIVKKFQRQKKTRV-RTLGGSSFITSSIFRMLHKP       5048
YP_009505581.1        NA-LPEDFSDRLLDTASRTIMPFSTKIVTKHQRTKKPRV-RTIGGSSFITSSIFRMLHKP       5353
YP_007697636.1        RT-LYGEYRDKLVYHKRHSADQALTLVINKVAISTKHRD-RTILAININKSEAGRSLFRW       3157
YP_007697642.1        RQ-LYGSARDDLVYHKRHSADQMLTLVINKVANSTKHRD-RTILAININKSEPGRALYRW       3184
YP_009026378.1        RE-LLGKYRDAIVYHKRHSADQQLTLTINKVAPSKNHRD-RTILAISINKSEPGRSLYRW       3163
AEH26445.1            RE-LYGNYRDAIVYHKRHSADQQLTLTINKVAPSKNHRD-RTILAISINKSEPGRSLYRW       3183
YP_007697629.1        RE-LLGDYRDAIVHHKRHSADQHLTLTINKVATSTKHRD-RTILAISINKSEAGRSLYRW       3187
EAV_nsp9              ---QSYCLIDDMVSQSMKSNLQTATMATCKRQYCSKYKI-RSILGTNNYIGLGLRACLSG       409
ABI64079.1            ---QSYCLIDDMVSQSMKSNLQTATMATCKRQYCSKYKI-RSILGTNNYIGLGLRACLSG       359
AEC48047.1            ---QSIPNINSICEQAVSEVWQSVTPVTLKKQYCSKPKT-RTILGTNGLISLGLRALLSG       358
YP_009109556.3        ---QALPDIDELCEKAIAEVWQTVTPVTLKKQFCSKAKT-RTILGTNAMASLALRALLSG       2461
AZT89154.1            ---QALPDIDELCEKAIAEVWQTVTPVTLKKQFCSKAKT-RTILGTNAMASLALRALLSG       2461
AGA19090.1            ---QALPDINEICDRAAKEVWQSVTPVTLKKQFCSKFKT-RTILGTNALISLAIRAGLSG       359
AAA74104.1            ---QSHPDIDALCERACKEHWQTVTPCTLKKQYCSKAKT-RTILGTNNFVALGLRSALSG       355
AAA85664.1            ---QSHPDIDALCKRACEEHWQTVTPCTLKKQYCSKSKT-RTILGTNNFVALGLRSALSG       354
AEJ54658.1            ---QSIPEIDEVCARAVKENWQTVTPCTLKKQYCSKPKT-RTILGTNNFIALAHRSALSG       361
QPK93580.1            ---QSVPEIDVLCAQAVRENWQTVTPCTLKKQYCGKKKT-RTILGTNNFVALAHRAALSG       2728
ANT45956.1            ---QSVPEIDVLCAQAVRENWQTVTPCTLKKQYCGKKKT-RTILGTNNFVALAHRAALSG       2728
ALL55209.1            ---QSIPEIDVLCAQAVRENWQTVTPCTLKKQYCGKKKT-RTILGTNNFVALAHRAALSG       2759
APU51031.1            ---QSVPEIDVLCAQAVRENWQTVTPCTLKKQYCGKKKT-RTILGTNNFIALAHRAVLSG       2678
AFP43966.1            ---QSVPEIDVLCAQAVRENWQTVTPCTLKKQYCGKKKT-RTILGTNNFIALAHRAALSG       2859
YP_005352880.1        YD-MSYAEQNQLFEYTKRNVLPTLTQMNLKYAISAKDRA-RTVAGVSIVSTMTNRQYHQK       4095
YP_002308505.1        YD-MTYVEQNQLFEYTKRNVLPTLTQMNLKYAISAKDRA-RTVAGVSIISTMTNRQYHQK       4233
YP_005352845.1        YD-MTHAEQNQLFEYTKRNVLPTLTQMNLKYAISAKDRA-RTVAGVSIISTMTNRQYHQK       4202
YP_002308478.1        YD-MTYAEQNQLFEYTKRNVLPTLTQMNLKYAISAKDRA-RTVAGVSIISTMTNRQYHQK       4149
ABG47051.1            YESMSYQEQDELFAVTKRNVLPTITQMNLKYAISAKNRA-RTVAGVSILSTMTNRQYHQK       5003
YP_009513008.1        YESMSFQEQDELFALTKRNVVPTITQMNLKYAISAKNRA-RTVAGVSILSTMTNRQYHQK       5016
ABN10874.1            YESLSYQEQDELFAMTKRNVLPTITQMNLKYAISAKNRA-RTVAGVSILSTMTNRQYHQK       5043
YP_009047202.1        YESMSYQEQDELFAMTKRNVIPTMTQMNLKYAISAKNRA-RTVAGVSILSTMTNRQYHQK       4952
AAX76519.1            YEALSFEEQNEIYAYTKRNVLPTLTQMNLKYAISAKNRA-RTVAGVSILSTMTGRMFHQK       4997
AFE48810.1            YEALSFDEQDDIYAYTKRNVLPTLTQMNLKYAISAKNRA-RTVAGVSILSTMTGRMFHQK       4999
AAF19384.1            YEALSFEEQDEVYAYTKRNVLPTLTQMNLKYAISAKNRA-RTVAGVSILSTMTGRMFHQK       581
YP_009019180.1        YETLSYEEQDELFATTKRNVLPTMTQMNLKYAISGKARA-RTVGGVSLLSTMTTRQYHQK       4570
ACT10947.1            YETLSYEEQDALFALTKRNVLPTMTQMNLKYAISGKARA-RTVGGVSLLSTMTTRQYHQK       4581
YP_001718610.1        YESLSYEEQDALYALTKRNVLPTMTQLNLKYAISGKERA-RTVGGVSLLSTMTTRQFHQK       4783
YP_001718603.1        YEALSYEEQDALYAVTKRNILPTMTQLNLKYAISGKERA-RTVGGVSLLSTMTTRQFHQK       4820
YP_008439200.1        YESLSYEEQDELYALTKRNILPTMTQLNLKYAISGKERA-RTVGGVALLSTMTTRQFHQK       4712
AFU92112.1            YESLTYEEQDALYAVTKRNVLPTMTQLNLKYAISGKERA-RTVGGVSLLSTMTTRQYHQK       4667
YP_001351683.1        YDSLSYEEQDDLYAYTKRNIIPTMTQLNLKYAISGKDRA-RTVGGVSLLSTMTTRQYHQK       4680
AGK89913.1            YESLSYEEQDELYAYTKRNILPTMTQLNLKYAISGKDRA-RTVGGVSLLSTMTTRQYHQK       4669
YP_003766.2           YESISYEEQDALFALTKRNVLPTMTQLNLKYAISGKERA-RTVGGVSLLSTMTTRQYHQK       4612
AFI49429.1            YESISYEEQDAMFALTKRNILPTMTQLNLKYAISGKERA-RTVGGVSLLATMTTRQFHQK       4641
YP_001552234.1        YETFSYEEQDALYAMTKRNILPTMTQLNLKYSISGKARA-RTVGGVSLLATMTTRQFHQK       4611
AGK85497.1            YE-MSLEEQDQLFESTKKNVLPTITQMNLKYAISAKNRA-RTVAGVSILSTMTNRQFNQK       4508
AHB63480.1            YESLSYAEQDQLFELTKRNILPTITQINMKYAISAKSRA-RTVAGVSILSTMTNRQFHQK       4515
ADM33581.1            YESLSYADQDELFAYTKRNVLPTITQMNLKYAISAKNRA-RTVAGVSIASTMTNRQFHQK       4782
AAP50483.1            YDSMSYEDQDALFAYTKRNVIPTITQMNLKYAISAKNRA-RTVAGVSICSTMTNRQFHQK       4943
SARS-CoV-2_nsp12      YDSMSYEDQDALFAYTKRNVIPTITQMNLKYAISAKNRA-RTVAGVSICSTMTNRQFHQK       574
YP_009724389.1        YDSMSYEDQDALFAYTKRNVIPTITQMNLKYAISAKNRA-RTVAGVSICSTMTNRQFHQK       4966
                                                   *       :  *::           *
```

```
YP_001661452.1        WLE-FTKT-RLSYNTVLIGFKDTHCGINKLINGI-KAGF---NP--KGKSKWISQDYPKF       4757
YP_009666324.1        WLE-FTKT-RLSYNTVLIGFKDTHCGINKLINGI-TAGF---NP--KGKAKWISQDYPKF       4769
YP_008798230.1        VTSKMVEAAQNSQGFCLIGVSKYGLKFSKFLKDK-YGSI--------EQFDVFGSDYTKC       4963
YP_337905.2           VTSKMVEVAQNSQGFCLIGVSKYGLKFSKFLKDK-YGAI--------EQFDVFGSDYTKC       4991
ABI97394.1            VTNKMVQTAQANIGPFLIGISKFNLGFHKYLSAHHPNGI--------EDCQVMGADYTKC       5100
YP_009505581.1        VTNKMVNTAQNNIGPFLIGVSKFNLGFHKYLTAHHPNGI--------EDCSVMGADYTKC       5405
YP_007697636.1        FLDKIKDT-ANKGGPILIGLIAQYGGWDKLYKNL-YKDSPIDNPDATEHVVLGGKDYPKW       3215
YP_007697642.1        HLDKIKYT-ANNGGPILIGFIAQYGGWDKFYKQL-YKNSPSDNPETAQYAVLGGKDYPKW       3242
YP_009026378.1        NLDKIKYT-ASLGGPILIGFTAQYGGWDKFYKQL-YKNSPADQPDIAEYAVLGGKDYPKW       3221
AEH26445.1            NLDKIKYT-SSLGGPILIGFTAQYGGWDKLYKYL-YKNSPADHPDIAEHAVLGGKDYPKW       3241
YP_007697629.1        NLDKIKYT-ASLGGPILIGFTAQYGGWDKFYKQL-YKNSPADQPGVAERAVLGGKDYPKW       3245
EAV_nsp9              VTAAFQKA-GKDGSPIYLGKSKFDPI---------PAP---------DK-YCLETDLESC       449
ABI64079.1            VTAAFQKA-GKDGSPIYLGKSKFDPI---------PAP---------DK-YCLETDLESC       399
AEC48047.1            VTARFQLA-GK-DSPICLGKSKFQRS---------DIRI--------TT-RCLETDLASC       398
YP_009109556.3        VTQGFQLA-GK-NSPICLGKSKFDPC---------TFEV--------KG-RCLETDLASC       2501
AZT89154.1            VTQGFQLA-GK-NSPICLGKSKFDPC---------TFEV--------KG-RCLETDLASC       2501
AGA19090.1            VTQAFQLA-GK-DTPICLGKSKYNPM---------QVYP--------QG-VCMETDLASC       399
AAA74104.1            VTQGFMRK-GI-GSPICLGKNKFTPL---------PTKV--------SG-RCLEADLASC       395
AAA85664.1            VTQGFMRK-GI-GTPICLGKNKFTPL---------PVRI--------GG-RCLEADLASC       394
AEJ54658.1            VTQAFMKK-AW-KSPIALGKNKFKEL---------HCTV--------AG-RCLEADLASC       401
QPK93580.1            VTQGFMKK-AF-NSPIALGKNKFKEL---------QTPV--------LG-RCLEADLASC       2768
ANT45956.1            VTLGFMKK-AF-NSPIALGKNKFKEL---------QTPV--------LG-RCLEADLASC       2768
ALL55209.1            VTQGFMKK-AF-NSPIALGKNKFKEL---------QTPV--------LG-RCLEADLASC       2799
APU51031.1            VTQGFMKK-AF-NSPIALGKNKFKEL---------QTPV--------LG-RCLEADLASC       2718
AFP43966.1            VTQGFMKK-AF-NSPIALGKNKFKEL---------QTPV--------LG-RCLEADLASC       2899
YP_005352880.1        ILK-S-IS-LARNQTIVIGTTKFYGGWDNMLRKL-MNGI--------KNPTLAGWDYPKC       4143
YP_002308505.1        ILK-S-IS-LARNQTIVIGTTKFYGGWDNMLRRL-MHNI--------NNPILVGWDYPKC       4281
YP_005352845.1        MLK-X-IS-LARNQTIVIGTTKFYGGWDNMLRRL-MCNI--------NNPILVGWDYPKC       4250
YP_002308478.1        MLK-S-IS-LARNQTIVIGTTKFYGGWDNMLRRL-MNGI--------NNPILVGWDYPKC       4197
ABG47051.1            MLK-S-MA-ATRGATCVIGTTKFYGGWDFMLKTL-YKDV--------ESPHLMGWDYPKC       5051
YP_009513008.1        MLK-S-MA-ATRGATCVIGTTKFYGGWDFMLKTL-YKDV--------DNPELMGWDYPKC       5064
ABN10874.1            MLK-S-MA-ATRGSTCVIGTTKFYGGWDFMLKTL-YKDV--------DNPHLMGWDYPKC       5091
YP_009047202.1        MLK-S-MA-ATRGATCVIGTTKFYGGWDFMLKTL-YKDV--------DNPHLMGWDYPKC       5000
AAX76519.1            CLK-S-IA-ATRGVPVVIGTTKFYGGWDDMLRHL-IKDV--------DNPVLMGWDYPKC       5045
AFE48810.1            CLK-S-IA-ATRGVPVVIGTTKFYGGWDDMLRRL-IKDV--------DNPVLMGWDYPKC       5047
AAF19384.1            CLK-S-IA-ATRGVPVVIGTTKFYGGWDDMLRRL-IKDV--------DSPVLMGWDYPKC       629
YP_009019180.1        HLK-S-IA-ATRNATVVIGTTKFYGGWDDMLKNL-MRDV--------DNGCLMGWDYPKC       4618
ACT10947.1            HLK-S-IA-ATRNATVVIGTTKFYGGWDNMLKNL-MRDV--------DNGCLMGWDYPKC       4629
YP_001718610.1        HLK-S-IV-NTRNATVVIGTTKFYGGWDNMLRNL-MDGV--------DNACLMGWDYPKC       4831
YP_001718603.1        HLK-S-IV-NTRNATVVIGTTKFYGGWDNMLRNL-IDGV--------DNACLMGWDYPKC       4868
YP_008439200.1        HLK-S-IV-NTRGASVVIGTTKFYGGWDAMLKTL-IHGV--------ENPHLMGWDYPKC       4760
AFU92112.1            HLK-S-IV-NTRNASVVIGTTKFYGGWDNMLNTL-INGV--------ENPCLMGWDYPKC       4715
YP_001351683.1        HLK-S-IV-NTRGASVVIGTTKFYGGWDNMLKTL-IKDV--------ENPHLMGWDYPKC       4728
AGK89913.1            HLK-S-IV-NTRGASVVIGTTKFYGGWDNMLKNL-IDGV--------ENPCLMGWDYPKC       4717
YP_003766.2           HLK-S-IV-NTRNATVVIGTTKFYGGWNNMLRTL-IDGV--------ENPMLMGWDYPKC       4660
AFI49429.1            CLK-S-IV-ATRNATVVIGTTKFYGGWDNMLKNL-IADV--------DDPKLMGWDYPKC       4689
YP_001552234.1        HLK-S-IV-NTRNAPVVIGTTKFYGGWDNMLKNL-MNDV--------DNGALMGWDYPKC       4659
AGK85497.1            ILK-S-IV-NTRNAPVVIGTTKFYGGWDNMLRNL-IQGV--------EDPILMGWDYPKC       4556
AHB63480.1            CLK-S-IV-NTRNATVVIGTTKFYGGWDNMLRNL-MRGV--------EDPVLMGWDYPKC       4563
ADM33581.1            MLK-S-IA-AARGASVVIGTTKFYGGWNRMLRTL-CEGV--------DNPHLMGWDYPKC       4830
AAP50483.1            LLK-S-IA-ATRGATVVIGTSKFYGGWHNMLKTV-YSDV--------ETPHLMGWDYPKC       4991
SARS-CoV-2_nsp12      LLK-S-IA-ATRGATVVIGTSKFYGGWHNMLKTV-YSDV--------ENPHLMGWDYPKC       622
YP_009724389.1        LLK-S-IA-ATRGATVVIGTSKFYGGWHNMLKTV-YSDV--------ENPHLMGWDYPKC       5014
                                       :*                                  *  .
```

```
YP_001661452.1        DTCVDTMAQYSYIVNH-AYHYT-------HTNLSLIVRGLCQLIANSTSPIIYYNSILIR       4809
YP_009666324.1        DTCVDTMAQYSYIMNH-AYHYT-------HTNLSLITRGLCQLIANSTSPIIYYNSMLIR       4821
YP_008798230.1        DRTFPLSFRALTAALLYELG----------GWEE-ESWLYLNEVNSYMLDTMICDGMLLN       5012
YP_337905.2           DRTFPLSFRALTAALLYELG----------GWEE-DSWLYLNEVNSYMLDTMLCDGMLLN       5040
ABI97394.1            DRSFPVVCRALSAALFYELG----------HLEP-NNHWFLNEMFAFLLDPSFISGHIFN       5149
YP_009505581.1        DRSFPVVCRALSAALFYDLG----------NLPH-KSHWFINECFAFIFDQSYIAGHVFN       5454
YP_007697636.1        DRRISNMLQLTTTNVFFNLIDQNTQFKNNQASPSETWHEYMSETTQILFDYLVFGNELYQ       3275
YP_007697642.1        DRRISNMLQLTTTNVLFSLIDQTTQLRRNNANPAETWHEYMAETTQVLFDYLVFGNELHQ       3302
YP_009026378.1        DRRISNMLQLTTTTVLYSLIDPNTQTKLNNATPSQTWHEYMAETTQVLFDYLVFGNELYQ       3281
AEH26445.1            DRRISNMLQLTTTTVLYSLIDPNTQSKLNNATPAQTWHEYMAETTQVLFDYLVFGNELYQ       3301
YP_007697629.1        DRRISNMLQLTTTTILYSLIDPNTQKKLNNANPAQTWHEYMAETTQVLFDYLVFGNELYQ       3305
EAV_nsp9              DRSTPALVRWFATNLIFEL--A-------GQPELVHSYVL-NCCH----DLVVAGSVAFT       495
ABI64079.1            DRSTPALVRWFATNLIFEL--A-------GQPELVHSYVL-NCCH----DLVVAGSVAFT       445
AEC48047.1            DRSTPALVRYFSTRLLFEL--A-------CAERAIPLYVA-NCCH----DLLVTQTSAVT       444
YP_009109556.3        DRSTPAIVRHFATKLLFEM--A-------CAERALPLYVV-NCCH----DLIVTQTSAAT       2547
AZT89154.1            DRSTPAIVRHFATKLLFEM--A-------CAERALPLYVV-NCCH----DLIVTQTSAAT       2547
AGA19090.1            DRSTPAVVRWFATELLFEL--G-------CCSHLKPLYIA-NCCH----DLLVTQTTACT       445
AAA74104.1            DRSTPAIIRWFTTNLLFEL--A-------GPEEWIPSYVL-NCCH----DAVSTMSGCFD       441
AAA85664.1            DRSTPAIIRWFTTNLLFEL--A-------GAEEWIPSYVL-NCCH----DVVSTMSGCFD       440
AEJ54658.1            DRSTPAIVRWFVANLLYEL--A-------GCEEYLPSYVL-NCCH----DLVATQDGAFT       447
QPK93580.1            DRSTPAIVRWFAAHLLYEL--A-------CAEDHLPSYVL-NCCH----DLLVTQSGAVT       2814
ANT45956.1            DRSTPAIVRWFAAHLLYEL--A-------CAEDHLPSYVL-NCCH----DLLVTQSGAVT       2814
ALL55209.1            DRSTPAIVRWFAAHLLYEL--A-------CAEEHLPSYVL-NCCH----DLLVTQSGAVT       2845
APU51031.1            DRSTPAIVRWFAANLLYEL--A-------CAEEHLPSYVL-NCCH----DLLVTQSGAVT       2764
AFP43966.1            DRSTPAIVRWFAANLLYEL--A-------CAEEYLPSYVL-NCCH----DLLVTQSGAVT       2945
YP_005352880.1        DRSMPNILRIAASCLLARKH-T-------CCNQSQRFYRLANECCQVLSEVVVSGNNLYV       4195
YP_002308505.1        DRSMPNILRIASSCLLARKH-T-------CCNQSQRFYRLANECCQVLSEVVVSGNNLYV       4333
YP_005352845.1        DRSMPNMLRIAASCLLARKH-T-------CCNQSQRFYRLANECCQVLSEVVVSGNNLYV       4302
YP_002308478.1        DRSMPNMLRIAASCLLARKH-T-------CCNQSQRFYRLANECCQVLSEVVVSGNNLYV       4249
ABG47051.1            DRAMPNMCRILASLILARKHST-------CCTNSDRFYRLANECAQVLSEYVLCGGGYYV       5104
YP_009513008.1        DRAMPNMCRIFASLVLARKHST-------CCTNSDRFYRLANECAQVLSEYVLCGGGYYV       5117
ABN10874.1            DRAMPNMCRIFASLILARKHST-------CCTNTDRFYRLANECAQVLSEYVLCGGGYYV       5144
YP_009047202.1        DRAMPNMCRIFASLILARKHGT-------CCTTRDRFYRLANECAQVLSEYVLCGGGYYV       5053
AAX76519.1            DRAMPNILRIVSSLVLARKHEF-------CCSHGDRFYRLANECAQVLSEIVMCGGCYYV       5098
AFE48810.1            DRAMPNILRIVSSLVLARKHDA-------CCTQSDRFYRLANECAQVLSEIVMCGGCYYV       5100
AAF19384.1            DRAMPNILRIISSLVLARKHDS-------CCSHTDRFYRLANECAQVLSEIVMCGGCYYV       682
YP_009019180.1        DRALPNMIRMASAMILGSKHVG-------CCTHSDRFYRLSNELAQVLTEVVHCTGGFYI       4671
ACT10947.1            DRALPNMIRMASAMILGSKHIG-------CCTHSDRFYRLSNELAQVLTEVVHCTGGFYI       4682
YP_001718610.1        DRALPNMIRMISAMILGSKHVN-------CCTNSDRYYRLCNELAQVLTEVVYSNGGFYM       4884
YP_001718603.1        DRALPNMIRMISAMILGSKHEN-------CCTNSDRYYRLCNELAQVLTEVVYSNGGFYL       4921
YP_008439200.1        DRALPNMVRMISAMILGSKHVT-------CCTASDRFFRLANELAQVLTEVVYSNGGFYL       4813
AFU92112.1            DRALPNMIRMISAMILGSKHTT-------CCTTDERYYRLCNELAQVLTEVVYSNGGFYF       4768
YP_001351683.1        DRALPNMIRMISAMILGSKHVN-------CCSSSDRYYRLCNELAQVLTEMVYSNGGFYV       4781
AGK89913.1            DRALPNMIRMISAMILGSKHTT-------CCSSTDRFFRLCNELAQVLTEVVYSNGGFYL       4770
YP_003766.2           DRALPNMIRMISAMVLGSKHVN-------CCTATDRFYRLGNELAQVLTEVVYSNGGFYF       4713
AFI49429.1            DRAMPSMIRMLSAMILGSKHVT-------CCTASDKFYRLSNELAQVLTEVVYSNGGFYF       4742
YP_001552234.1        DRAMPSMIRMLAAMVLGSKHVT-------CCTDSDRFYRLSNELAQVLNEVVHSNGGFYV       4712
AGK85497.1            DRAMPNLLRIAASLVLARKHTN-------CCTWSERIYRLYNECAQVLSETVLATGGIYV       4609
AHB63480.1            DRAMPSLLRLSASLILARRHKG-------CCDWNERIYRLANEAAQVLSEVALSNGGLYV       4616
ADM33581.1            DRAMPNLLRIFASLILARKHAT-------CCNASERFYRLANECAQVLSEMVLCGGGFYV       4883
AAP50483.1            DRAMPNMLRIMASLVLARKHNT-------CCNLSHRFYRLANECAQVLSEMVMCGGSLYV       5044
SARS-CoV-2_nsp12      DRAMPNMLRIMASLVLARKHTT-------CCSLSHRFYRLANECAQVLSEMVMCGGSLYV       675
YP_009724389.1        DRAMPNMLRIMASLVLARKHTT-------CCSLSHRFYRLANECAQVLSEMVMCGGSLYV       5067
                      *       :
```

```
YP_001661452.1       KLHGVSSGDGATAIKNSHCNSVITNIAFYRQIVDNQV-----PEEYRGLQSTLYTTLING       4864
YP_009666324.1       KLHGVSSGDGATAIKNSHCNSIITNIAFYRQIIDNQV-----PEEYRGLQSTLYNTLING       4876
YP_008798230.1       KPGGTSSGDATTAHSNTFYNYMVHYVVAFKTILS-DLTES-----NRVM-RVTAHNAYTT       5065
YP_337905.2          KPGGTSSGDATTAHSNTFYNYMVHYVVAFKTILS-DLSDC-----NKVM-RIAAHNAYTT       5093
ABI97394.1           KPGGTTSGDSTTAFSNSFYNYFVHLYIQYLTFLTTEMPPS-----YQPL-CNLAHQAFST       5203
YP_009505581.1       KPGGTTSGDSTTAFSNSFYNYFVHLFIQFQTFLSADLPDS-----LKPI-QALAHRAYTL       5508
YP_007697636.1       KPGGVTSGNSRTADGNSFLHMLIDMYALIIQLIQSTPENVHIE---SKLRNTLCKTVFET       3332
YP_007697642.1       KPGGVTSGNSRTADGNSLLHLLIDFYAIIIQLIQSTPENVDLQ---SELRSRLCKIVFTK       3359
YP_009026378.1       KPGGVTSGNSRTADGNSLLHLLIDFYAIIIQLIQSTPDNVHLE---PELRNKLCKTVFTR       3338
AEH26445.1           KPGGVTSGNSRTADGNSLLHLLIDFYAIISQLIQSTPENVHLE---VNLRNALCKTVFTK       3358
YP_007697629.1       KPGGVTSGNSRTADGNSLLHLLIDFYAIITQLIQSKPHNVHLH---SKLRNRLCKTVFTK       3362
EAV_nsp9             KRGGLSSGDPITSISNTIYSLVLYTQHMLLCGLEGY----FPEIAEKYLDGSLE------       545
ABI64079.1           KRGGLSSGDPITSISNTIYSLVLYTQHMLLCGLEGY----FPEIAEKYLDGSLE------       495
AEC48047.1           KRGGLSSGDPITSIANTIYSLLLYTQHMVLSAFKIG----H-PIALAFMQRRLT------       493
YP_009109556.3       KRGGLSSGDPVTSIANTIYSLVLYVQHMVLTLLENG----H-PLSLKFLSGKLN------       2596
AZT89154.1           KRGGLSSGDPVTSIANTIYSLVLYVQHMVLTLLENG----H-PLSLKFLSGKLN------       2596
AGA19090.1           KRGGLSSGDPVTSISNTIYSLILYTQHMILSALKEG----H-KVALKYIEGRLT------       494
AAA74104.1           KRGGLSSGDPVTSVSNTVYSLVIYAQHMVLSAFRCG----H-KVGGLFLRDSLE------       490
AAA85664.1           KRGGLSSGDPVTSISNTVYSLIIYAQHMVLSAFRCG----H-KIGGLFLQDSLE------       489
AEJ54658.1           KRGGLSSGDPVTSVSNTVYSLIIYAQHMVLSALKMG----H-EIGLKFLEEQLK------       496
QPK93580.1           KRGGLSSGDPITSVSNTIYSLVIYAQHTVLSYFKSG----H-PHGLLFLQDQLK------       2863
ANT45956.1           KRGGLSSGDPITSVSNTIYSLVIYAQHMVLSYFKSG----H-PHGLLFLQDQLK------       2863
ALL55209.1           KRGGLSSGDPITSVSNTIYSLVIYAQHMVLSYFKSG----H-PHGLLFLQDQLK------       2894
APU51031.1           KRGGLSSGDPITSVSNTIYSLVIYAQHMVLSYFKSG----H-PHGLLFLQDQLK------       2813
AFP43966.1           KRGGLSSGDPITSVSNTIYSLVIYAQHMVLSYFKSG----H-PHGLLFLQDQLK------       2994
YP_005352880.1       KPGGTSSGDATTAYANSVFNILQVVTANVAAFLSTS-TTSHSLKEVADLHRNLYEDIYRG       4254
YP_002308505.1       KPGGTSSGDATTAYANSVFNILQVVSANVAAFLSTS-TTSHSNNDIACLHRALYEDIYRG       4392
YP_005352845.1       KPGGTSSGDATTAYANSVFNILQVVSANVAAFLSTS-TTTHLNKDIADLHRSLYEDIYRG       4361
YP_002308478.1       KPGGTSSGDATTAYANSVFNILQVVSANVATFLSTS-TSSHNSREIADLHRNLYEDIYRG       4308
ABG47051.1           KPGGTSSGDATTAYANSVFNILQATTANVSALMSAN-GNTIIDREIKDMQFDLYINVYRK       5163
YP_009513008.1       KPGGTSSGDATTAYANSVFNILQAVTSNVSALMSAN-GNKIIDKEVKDMQFELYVNVYRN       5176
ABN10874.1           KPGGTSSGDATTAYANSVFNILQATTANVSALMGAN-GNTIVDEEVKDMQFELYVNVYRK       5203
YP_009047202.1       KPGGTSSGDATTAYANSVFNILQATTANVSALMGAN-GNKIVDKEVKDMQFDLYVNVYRS       5112
AAX76519.1           KPGGTSSGDATTAFANSVFNICQAVTANVCSLMACN-GHKIEDLSIRNLQKRLYSNVYRT       5157
AFE48810.1           KPGGTSSGDATTAFANSVFNICQAVSANVCSLMACN-GNKIEDLSIRALQKRLYSHVYRS       5159
AAF19384.1           KPGGTSSGDATTAFANSVFNICQAVSANVCSLMACN-GHKIEDLSIRELQKRLYSNVYRA       741
YP_009019180.1       KPGGTTSGDGTTAYANSAFNIFQAVSANVNRLLSVD-SNTCNNYNVKALQRKIYDNCYRS       4730
ACT10947.1           KPGGTTSGDGTTAYANSAFNIFQAVSANVNKLLGVD-SNTCNNVTVKSIQRKIYDNCYRS       4741
YP_001718610.1       KPGGTTSGDATTAYANSVFNIFQAVSANINRILGIN-SNTCNNLAVKSLQRMLYDNCYRS       4943
YP_001718603.1       KPGGTTSGDATTAYANSVFNIFQAVSANINRILGVN-SNTCNNLTVKELQRSLYDNCYRT       4980
YP_008439200.1       KPGGTTSGDATTAYANSVFNIFQAVSANVNRLLRVD-SNLCNNLDVRDLQRRLYECCYRS       4872
AFU92112.1           KPGGTTSGDASTAYANSVFNIFQAVSSNINRLLTID-SNVCNNVSVKTLQRELYDNCYRS       4827
YP_001351683.1       KPGGTTSGDATTAYANSVFNIFQATSANVNRLLSVD-SNTCNNIEVKQLQRKLYDCCYRS       4840
AGK89913.1           KPGGTTSGDATTAYANSVFNIFQAVSANVNKLLSVD-SNVCHNLEVKQLQRKLYDCCYRS       4829
YP_003766.2          KPGGTTSGDASTAYANSIFNIFQAVSSNINRLLSVP-SDSCNNVNVRDLQRRLYDNCYRL       4772
AFI49429.1           KPGGTTSGDATTAYANSVFNIFQAVSSNINRILSVN-SSNCNNLNVKKLQKQLYDNCYRN       4801
YP_001552234.1       KPGGTTSGDATTAYANSVFNIFQAVSSNINRLLSVD-SNVCNNLYVKKLQRSIYDNCYRS       4771
AGK85497.1           KPGGTSSGDATTAYANSVFNIIQATSANVARLLSVI-TRDIVYDDIKDLQYELYQQVYRR       4668
AHB63480.1           KPGGTSSGDATTAYANSAFNLFQATAANVAQLLATP-TSRIYVEEVRALQHELYTQVYRR       4675
ADM33581.1           KPGGTSSGDSTTAYANSVFNICQAVSANLNTFLSID-GNKIYTTYVQDLQRRLYLGIYRS       4942
AAP50483.1           KPGGTSSGDATTAYANSVFNICQAVTANVNALLSTD-GNKIADKYVRNLQHRLYECLYRN       5103
SARS-CoV-2_nsp12     KPGGTSSGDATTAYANSVFNICQAVTANVNALLSTD-GNKIADKYVRNLQHRLYECLYRN       734
YP_009724389.1       KPGGTSSGDATTAYANSVFNICQAVTANVNALLSTD-GNKIADKYVRNLQHRLYECLYRN       5126
                     *  * :**:  *:  *:               .               :
```

```
YP_001661452.1        IHSK-------------DDAYSTHRAFEWNISRCATLSDDTLAIINPDVLD---------       4902
YP_009666324.1        IQSK-------------DDAYSTHRAFEWNISRCATLSDDTLAIINPDVFD---------       4914
YP_008798230.1        GNY---D-----------VFNTLLEEQFQTNYFLNFLSDDSFIFSEPGALKIFTC---EN       5108
YP_337905.2           GDY---G-----------VFNTLLEEQFQTNYFLNFLSDDSFIFSKPGALKIFTC---EN       5136
ABI97394.1            GNTETYD-----------LYFSMADDLNSTEYFLHFLSDDSFIISKPTAFPIFTP---AN       5249
YP_009505581.1        GEPDTYD-----------LYFSQIDDLNSTQYFLHFLSDDSFIISKPEAFPIFTP---AN       5554
YP_007697636.1        IPSDYIDNTNVNLLRNTDILHRIRTR----IAKGAYLSDDGLILIDTRLVK---------       3379
YP_007697642.1        IPADYIDDLNTDRLVKTDVLKTIRLN----VAKGLYLSDDGLLVFDPRIIR---------       3406
YP_009026378.1        IPSDYIDVSN-VTLRNTDVLRTIRLR----VAKGLYLSDDGLIVIDPRIIR---------       3384
AEH26445.1            IPSDYIDSSC-VTLRNTDILHTIRRR----VAKGAYLSDDGLIVIDPRIIR---------       3404
YP_007697629.1        IPADYINTSC-VTLRKTDTLRTIRLK----IAKNLVLSDDGLLVLDPEIID---------       3408
EAV_nsp9              --------------------LRDMFK----YVRVYIYSDDVVL-----------------       564
ABI64079.1            --------------------LRDMFK----YVRVYIYSDDVVL-----------------       514
AEC48047.1            --------------------MEDLIK----VQRFVVYSDDLVL-----------------       512
YP_009109556.3        --------------------FQDLYK----LQAFIVYSDDLIL-----------------       2615
AZT89154.1            --------------------FQDLYK----LQAFIVYSDDLIL-----------------       2615
AGA19090.1            --------------------LEDLIA----VQPFVVYSDDLVL-----------------       513
AAA74104.1            --------------------MEQLFE----LQPLLVYSDDVVL-----------------       509
AAA85664.1            --------------------MEQLFE----LQPLLVYSDDVVF-----------------       508
AEJ54658.1            --------------------FEDLLE----IQPMLVYSDDLVL-----------------       515
QPK93580.1            --------------------FEDMLK----VQPLIVYSDDLVL-----------------       2882
ANT45956.1            --------------------LEDMLK----VQPLIVYSDDLVL-----------------       2882
ALL55209.1            --------------------FEDMLK----VQPLIVYSDDLVL-----------------       2913
APU51031.1            --------------------FEDMLK----VQPLIVYSDDLVL-----------------       2832
AFP43966.1            --------------------FEDMLK----VQPLIVYSDDLVL-----------------       3013
YP_005352880.1        DSNN---KTVID------NFYDHLAT----YFGLMILSDDGVACIDTEAAANGVVADLNG       4301
YP_002308505.1        DSSD---TAVIN------SFYHHLQT----YFGLMILSDDGVACIDSDAAKQGSVADLDG       4439
YP_005352845.1        DSND---ITVID------RFYQHLQS----YFGLMIFSDDGVACIDSDVAKSGAVADLDG       4408
YP_002308478.1        DSNN---TTIID------QFYQHLQK----YFGLMILSDDGVACIDTEAAASGVVSNLDG       4355
ABG47051.1            VVPD---PKFVD------KYYAFLNK----HFSMMILSDDGVVCYNSDYAAKGYVASIQN       5210
YP_009513008.1        SKPD---FKFVD------KYYAFLNR----HFSMMILSDDGVVCYNKDYAARGYIAGIQN       5223
ABN10874.1            SQPD---PKFVD------RYYAFLNK----HFSMMILSDDGVVCYNSDYATKGYIASIQN       5250
YP_009047202.1        TSPD---PKFVD------KYYAFLNK----HFSMMILSDDGVVCYNSDYAAKGYIAGIQN       5159
AAX76519.1            DYVD---YTFVN------EYYEFLCK----HFSMMILSDDGVVCYNSDYASKGYIANISV       5204
AFE48810.1            DTVD---PTFVT------EYYEFLNK----HFSMMILSDDGVVCYNSDYASKGYIANISA       5206
AAF19384.1            DHVD---PAFVN------EYYEFLNK----HFSMMILSDDGVVCYNSEFASKGYIANISA       788
YP_009019180.1        SVVD---PLVID------EYYAYLRK----HFSMMILSDDGVVCYNKEYADLGYVADISA       4777
ACT10947.1            SSVD---DDFVV------EYFSYLRK----HFSMMILSDDGVVCYNKDYADLGYVADIGA       4788
YP_001718610.1        SAVD---PGFVD------TFYGYLRK----HFSMMILSDDGVVCYNKEYASLGYVADINA       4990
YP_001718603.1        STVD---PAFVD------TFYGYLRK----HFSMMILSDDGVVCYNKEYASLGYVADIGA       5027
YP_008439200.1        STVD---DNFVN------DYYGFLRK----HFSMMILSDDGVVCHNSEYAQLGYVADLNA       4919
AFU92112.1            SSVD---EQFID------KYYCYLRK----HFSMMILSDDGVVCYNKDYADLGYVADISA       4874
YP_001351683.1        SSVD---QSFVE------EYFGYLRK----HFSMMILSDDGVVCYNSEYAALGYVADLNA       4887
AGK89913.1            TTVD---DQFVV------EYYGYLRK----HFSMMILSDDGVVCYNNDYASLGYVADLNA       4876
YP_003766.2           TSVE---ESFID------DYYGYLRK----HFSMMILSDDGVVCYNKDYAELGYIADISA       4819
AFI49429.1            SNVD---ESFVD------DFYGYLQK----HFSMMILSDDGVVCYNKIYAELGYIADISA       4848
YP_001552234.1        SAVD---DNVVT------DFYNYLKK----HFSMMILSDDGVVCYNKEYAALGYVGDISA       4818
AGK85497.1            VNFD---SAFVE------KFYSYLCK----NFSLMILSDDGVVCYNNTLAKQGLVADISG       4715
AHB63480.1            DKPD---MDFVY------TFYAYLNK----HFSLMILSDDGVVCYNSDYAEAGMVASIAS       4722
ADM33581.1            NTVD---YELVL------DYYNYLRK----HFSMMILSDDGVVCYNSDYAQKGYVADIQG       4989
AAP50483.1            RDVD---HEFVD------EFYAYLRK----HFSMMILSDDAVVCYNSNYAAQGLVASIKN       5150
SARS-CoV-2_nsp12      RDVD---TDFVN------EFYAYLRK----HFSMMILSDDAVVCFNSTYASQGLVASIKN       781
YP_009724389.1        RDVD---TDFVN------EFYAYLRK----HFSMMILSDDAVVCFNSTYASQGLVASIKN       5173
                                                           *** .
```

```
YP_001661452.1          --------LDQYLSSYRTLGGYEITNEKKIFVR----DEPYEFTFRYFFKE---------       4941
YP_009666324.1          --------LDQYLSSYRTLGGYEITNEKKIFVR----DEPYEFTSRYFFKE---------       4953
YP_008798230.1          FSNKLQTILHTKVDQTKSWSAS--------------GHIEEFCSAHIIKT---------       5144
YP_337905.2             FSNKLQTILHTKVDLTKSWATT--------------GHIEEFCSAHIIKT---------       5172
ABI97394.1              FSMKLQNVLGCYVDPAKSWSAD--------------GEIHEFCSSHICKI---------       5285
YP_009505581.1          FSIKLQSVLGCAVDVTKSWTEA--------------GKIHEFCSSHIELV---------       5590
YP_007697636.1          --------YDDFMAESHMISKYAIATNKHKYHLDPVQRKAREFLSQDTFHF---------       3422
YP_007697642.1          --------YDDFMAVSHMISYYMIAQNKHKYHIDAIERYAREFLSQDTIKF---------       3449
YP_009026378.1          --------YDDFMSISHLISHYMIAQNKHKYHIDAISRYAREFLSQDTIKF---------       3427
AEH26445.1              --------YDDFMSVSHLISHYMIAQNKHKYHIDAIQRYAREFLSQDTIKF---------       3447
YP_007697629.1          --------YTDYMSISHLISHYMMAQNKHKYHIDAISSYAREFLSQGPHKF---------       3451
EAV_nsp9                -----TTPNQHYAASFDRWVPH----------L-QALLGF-KVDPKKTVNTSSPSFLGCR       607
ABI64079.1              -----TTPNQHYAASFDRWVPH----------L-QALLGF-KVDPKKTVNTSSPSFLGCR       557
AEC48047.1              -----LNEPED-FPNFVYWSDH----------L-DLALGF-KTCRSKTVITTNPGFLGCR       554
YP_009109556.3          -----LNESDD-LPNFERWVPH----------L-ELALGF-KVDPKKTVITSNPGFLGCE       2657
AZT89154.1              -----LNESDD-LPNFERWVPH----------L-ELALGF-KVDPKKTVITSNPGFLGCE       2657
AGA19090.1              -----MQESPG-LPNFKYWNAH----------L-DLALGF-KTDPSKTVVTSKPSFLGCT       555
AAA74104.1              -----YDESSE-LPNYHFFVDH----------L-DLMLGF-KTDRSKTVITSDPQFPGCR       551
AAA85664.1              -----YNESDE-LPNYHFFVDH----------L-DLMLGF-KTDRSKTVITSEPKLPGCR       550
AEJ54658.1              -----YAEKPT-FPNYHWWVEH----------L-DLMLGF-KTDPKKTVITDKPSFLGCK       557
QPK93580.1              -----YAESPS-MPNYHWWVEH----------L-NLMLGF-QTDPKKTAITDSPSFLGCR       2924
ANT45956.1              -----YAESPS-MPNYHWWVEH----------L-NLMLGF-QTDPKKTAITDSPSFLGCR       2924
ALL55209.1              -----YAESPT-MPNYHWWVEH----------L-NLMLGF-QTDPKKTTITDSPSFLGCR       2955
APU51031.1              -----YAESPT-MPNYHWWVEH----------L-NLMLGF-QTDPKKTAITDSPSFLGCR       2874
AFP43966.1              -----YAESPT-MPNYHWWVEH----------L-NLMLGF-QTDPKKTAITDSPSFLGCR       3055
YP_005352880.1          FRDVLFYQNNVFMSDSKCWTET-----------DMTKGPHEFCSQHTVLT---------       4340
YP_002308505.1          FRDVLFYQNNVYMADSKCWTET-----------DMSVGPHEFCSQHTVLA---------       4478
YP_005352845.1          FRDILFYQNNVYMADSKCWTET-----------DMTVGPHEFCSQHTVLA---------       4447
YP_002308478.1          FRDILFYQNNVYMADSKCWTET-----------DMTVGPHEFCSQHTVLA---------       4394
ABG47051.1              FKETLYYQNNVFMSEAKCWVET-----------NLEKGPHEFCSQHTLYI---------       5249
YP_009513008.1          FKETLYYQNNVFLSEAKCWVEP-----------DIEKGPHEFCSQHTLLI---------       5262
ABN10874.1              FKETLYYQNNVFMSEAKCWVET-----------DLKKGPHEFCSQHTLFI---------       5289
YP_009047202.1          FKETLYYQNNVFMSEAKCWVET-----------DLKKGPHEFCSQHTLYI---------       5198
AAX76519.1              FQQVLYYQNNVFMSESKCWVEN-----------DITNGPHEFCSQHTMLV---------       5243
AFE48810.1              FQQVLYYQNNVFMSEAKCWVEN-----------DINHGPHEFCSQHTMLV---------       5245
AAF19384.1              FQQVLYYQNNVFMSEAKCWVET-----------DIEKGPHEFCSQHTMLV---------       827
YP_009019180.1          FKSVLYYQNNVFMSSAKCWVEP-----------DLSVGPHEFCSQHTMQI---------       4816
ACT10947.1              FKATLYYQNNVFMSTAKCWVEP-----------DLNVGPHEFCSQHTLQI---------       4827
YP_001718610.1          FKATLYYQNNVFMSTSKCWVEE-----------DLTKGPHEFCSQHTMQI---------       5029
YP_001718603.1          FKATLYYQNNVFMSTAKCWVEE-----------DLSKGPHEFCSQHTLQI---------       5066
YP_008439200.1          FKSVLYYQNNVFMSESKCWVEP-----------DVNKGPHEFCSQHTMQI---------       4958
AFU92112.1              FKATLYYQNNVFMSTAKCWVEP-----------DITKGPHEFCSQHTMQI---------       4913
YP_001351683.1          FKAVLYYQNNVFMSASKCWIEP-----------DINKGPHEFCSQHTMQI---------       4926
AGK89913.1              FKAVLYYQNNVFMSASKCWIEP-----------DINKGPHEFCSQHTMQI---------       4915
YP_003766.2             FKATLYYQNNVFMSTSKCWVEE-----------DLTKGPHEFCSQHTMQI---------       4858
AFI49429.1              FKATLYYQNGVFMSTAKCWTEE-----------DLSVGPHEFCSQHTMQI---------       4887
YP_001552234.1          FKATLYYQNNVFMSTAKCWVEE-----------DLSVGPHEFCSQHTMQI---------       4857
AGK85497.1              FREILYYQNNVYMADSKCWVEP-----------DLEKGPHEFCSQHTMLV---------       4754
AHB63480.1              FREVLFYQNNVFMADSKCWTEE-----------DVKIGPHEFCSQHSMLV---------       4761
ADM33581.1              FKELLYFQNNVFMSEAKCWVEP-----------DITKGPHEFCSQHTMLV---------       5028
AAP50483.1              FKAVLYYQNNVFMSEAKCWTET-----------DLTKGPHEFCSQHTMLV---------       5189
SARS-CoV-2_nsp12        FKSVLYYQNNVFMSEAKCWTET-----------DLTKGPHEFCSQHTMLV---------       820
YP_009724389.1          FKSVLYYQNNVFMSEAKCWTET-----------DLTKGPHEFCSQHTMLV---------       5212
                                                                 :
```

Supplementary Figure 19. Multiple sequence alignment of EAV nsp9. EAV nsp9 contains the RNA polymerase domain at approximately residue 360 to 549 that is conserved throughout nidoviruses. The arrow indicates the location of EAV nsp9 residue 380. “*” indicates complete conservation, “:” indicates strong conservation, and “.” indicates weaker conservation per the Clustal Omega algorithm. The EAV nsp9, EAV polyprotein (ABI64079.1), SARS-CoV-2 polyprotein (YP_009724389.1), and SARS-CoV-2 nsp12 sequences are included as separate entries in the alignment.

a

Score	Expect	Method	Identities	Positives	Gaps
37.4 bits(85)	7e-04	Compositional matrix adjust.	43/213(20%)	85/213(39%)	49/213(23%)

```
Query  360  DDMVSQSMKSNLQTATMATCKRQYCSKYKIRSILGTNNYIGLGLRACLSGVTAAFQK---  416
            D + + + ++ + T T    K    +K + R++ G         +  C +     F +
Sbjct  525  DALFAYTKRNVIPTITQMNLKYAISAKNRARTVAG--------VSICSTMTNRQFHQKLL  576

Query  417  ---AGKDGSPIYLGKSKF------------DPIPAPDKYCLETDLESCDRSTPALVRWFA  461
               A   G+ + +G SKF                +  P  + +  D   CDR+ P ++R  A
Sbjct  577  KSIAATRGATVVIGTSKFYGGWHNMLKTVYSDVENP--HLMGWDYPKCDRAMPNMLRIMA  634

Query  462  TNLI--------------FELAGQPELVHSYVLNCCHDLVVAGSVAFTKRGGLSSGDPIT  507
            + ++              + LA +   V S       ++V+ G    + K GG SSGD  T
Sbjct  635  SLVLARKHTTCCSLSHRFYRLANECAQVLS-------EMVMCGGSLYVKPGGTSSGDATT  687

Query  508  SISNTIYSLVLYTQHMLLCGLEGYFPEIAEKYL  540
            + +N+++++       +    L     +IA+KY+
Sbjct  688  AYANSVFNICQAVTANVNALLSTDGNKIADKYV  720
```

b

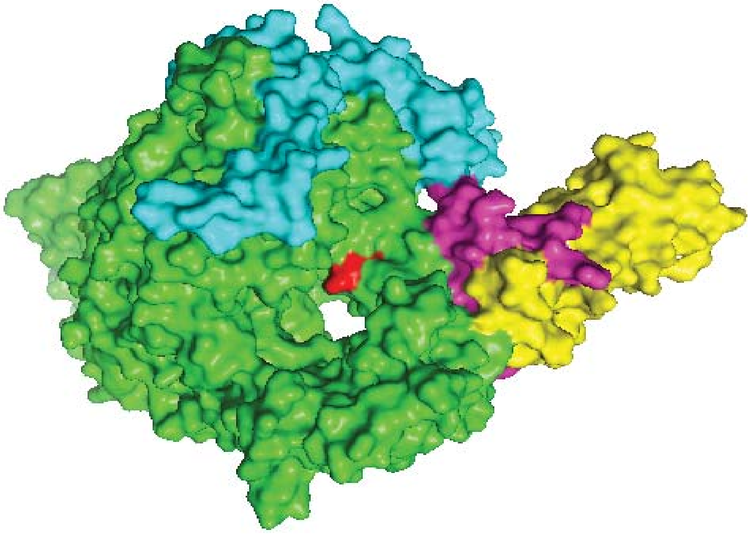

Supplementary Figure 20. Location of EAV nsp9 K380 residue in the SARS-CoV-2 nsp12/nsp8/nsp7 structure. a, Sequence alignment of EAV nsp9 (query) and SARS-CoV-2 nsp12 (subject) is shown as performed using the BLAST algorithm. b, *The homologous residue of EAV nsp9 K380 is K545 in SARS-CoV-2 nsp12. The cryo-EM structure of the SARS-CoV-2 nsp7 (magenta), nsp8 (two molecules in cyan and yellow), and nsp12 (green) heterocomplex is shown (PDB 7btf) as a surface representation. Residue K545 at the NTP binding site and at the entrance of the RNA tunnel is shown in red.

```
gi|72|EAV_nsp7        ------------------------------------------SLTATLA-ALTDDDFQFL        17
ABI64071.1            DVFSASGRFDRTFMMKYFLEGGVKESVTASVTRAYGKPITQESLTATLA-ALTDDDFQFL        1469
AEC48046.1            LLLSGNGSFDPAFFVRYVQEG-VREGVAS--------TIATESLSGALAVNLSAEELEFL        1822
AGA19089.1            SIISGTGSFDPAFLARYVHEG-IRQGVST--------GYATESLSACLATSLSKDELAFV        1812
YP_009109556.3        SVIGCHGSFDPTFLSRYVHEG-IRQGVSS--------GFGTESLSTALACALSEDELNFL        1861
AZT89154.1            SVIGCHGSFDPTFLSRYVHEG-IRQGVSS--------GFGTESLSTALACALSEDELNFL        1861
AAA74103.1            DMLVGNGCFDAAFFLKYFAEGNLRDGVSDSC------NMTPEGLTAALAITLSDDDLEFL        1932
AAA85663.1            DVLVGNGSFDAAFFLKYFAEGNLRDGVSDSC------NMTPEGLTAALAITLSDDDLEFL        1912
AEJ54657.1            NMLVGDGSFSSAFFLRYFAEGNLRKGVSQSC------GMNNESLTAALACKLSQADLDFL        2072
QPK93580.1            FVLVGDGAFSAAFFLRYFAEGKLREGVSQSC------GMNHESLTGALAMKLNDEDLDFL        2086
ANT45956.1            FVLVGDGAFSAAFFLRYFAEGKLREGVSQSC------GMNHESLTGALALKLSDEDLDFL        2086
ALL55209.1            YILVGDGVFSRAFFLRYFAEGKLREGVSQSC------GINHESLTGALAMKLDDEDLDFL        2117
APU51031.1            HILVGDGVFSAAFFLRYFAEGKLREGVSQSC------GMNHESLTGALAMRLNDEDLDFL        2036
AFP43966.1            NVLVGDGAFSAAFFLRYFAEGKLREGVSQSC------GMNHESLTGALAMRLNDEDLDFL        2217
                                                                .*:  **  *   :: *:

gi|72|EAV_nsp7        SDVLDCRAVRSAMNLRAALTSFQVAQYRNILNASLQVDRDAARSRRLMAKLADFAVE--Q        75
ABI64071.1            SDVLDCRAVRSAMNLRAALTSFQVAQYRNILNASLQVDRDAARSRRLMAKLADFAVE--Q        1527
AEC48046.1            NAIVPCKAFASASNLSKAMGDFQQSQAAKQLRRALAGVQATANTTAALATLDQFLSTKVR        1882
AGA19089.1            EQLVDCKAVVAAVNTQRALDDYILSTNAKRLRSHLTSVHATAAAQRALACLEDFLVGTSK        1872
YP_009109556.3        AQAVDHKAIVSAIHVHKTLQDYILSKNAKILRASLASVHANHNASKALASLDKFLQGTST        1921
AZT89154.1            AQAVDHKAIVSAIHVHKTLQDYILSKNAKILRASLASVHANHNASKALASLDKFLQGTST        1921
AAA74103.1            QRHSEFKCFVSASNMRNGAKEFIESAYARALRAQLAATDKIKASKSILAKLESFAGGVVT        1992
AAA85663.1            QRHSEFKCFVSASNMRNGAKEFIESAYARALRAQLAATDKIKASKSILAKLESFAGGVVT        1972
AEJ54657.1            SSLTNFKCFVSASNMKNAAGQYIEAAYAKALRQELASLVQIDKMKGVLSKLEAFAETATP        2132
QPK93580.1            TKLTDFKCFVSASNMRNAAGQFIEAAYAKALRTELAQLVQVDKVRGVLAKLEAFADTVAP        2146
ANT45956.1            TKWTDFKCFVSASNMRNAAGQFIEAAYAKALRVELAQLVQVDKVRGVMAKLEAFADTVAP        2146
ALL55209.1            TKLTDFKCFVSASNMRNAAGQFIEAAYAKALRVELAQLVQVDKVRGVLAKLEAFADTATP        2177
APU51031.1            MKWTDFKCFVSASNMRNAAGQFIEAAYAKALRVELAQLVQVDKVRGTLAKLEAFADTVAP        2096
AFP43966.1            TKWTDFKCFVSASNMRNAAGQFIEAAYAKALRIELAQLVQVDKVRGVLAKLEAFADTVAP        2277
                            :.. :* :      .:  :   . *.  *            :: *  *
                                                                           ↓
gi|72|EAV_nsp7        EVTAGDRVVVIDGLDR-----MAHFKDDLVLVPLTTKVVGGSRCTICDVVKEEANDTPVK        130
ABI64071.1            EVTAGDRVVVIDGLDR-----MAHFKDDLVLVPLTTKVVGGSRCTICDVVKEEANDTPVK        1582
AEC48046.1            GPRTGDTVVYLGKP-RGEIFDGYVGETQVILKPVRTQSVAGVTCTICEVTVPTEAMSK-H        1940
AGA19089.1            PLKPGDPVILLGAA-PGTISPAFCGDKEYVVRPIRSQTVAGTLCTLCQVEVVVEAGLL-G        1930
YP_009109556.3        QLKPGDPVILLGST-SAELVSVFSGDSEYIAEPIRSHPVAGTICTLCVVQAKCEGGLV-T        1979
AZT89154.1            QLKPGDPVILLGST-SAELVSVFSGDSEYIAEPIRSHPVAGTICTLCVVQAKCEGGLV-T        1979
AAA74103.1            QVEPGDVVVVLGKKVIGDLVEVVINDAKHVIRVIETRTMAGTQFSVGTICGDLENACE-D        2051
AAA85663.1            KVEPGDVVVVLGKKIVGDLVEITINDVKHVIRVIETRVMAGTQFSVGTICGDLENACE-D        2031
AEJ54657.1            SLDTGDVIVLLGQHPHGSILDINVGTERKTVSVQETRSLGGTKFSVCTVVSNTPVDAL-T        2191
QPK93580.1            QLLPGDIVVVLGHTPVGSIFDLKIGNTRHTLQSIETRLLAGSKMTVARVVDPTPTPPP-A        2205
ANT45956.1            QLSPGDIVVALGHTPVGSIFDLKVGNTKHTLQSIETRVLAGSKMTVARVVDPTPTPPP-A        2205
ALL55209.1            QLSPGDIVVALGHTPVGSIFDLKVGSTKHTLQAIETRVLAGSRMTVARVVDPTPTPPP-V        2236
APU51031.1            QLSPGDIVVALGHTPVGSIFDLKVGSTKHTLQAIETRVLAGSKMTVARVIHPTPTPPP-A        2155
AFP43966.1            QLSPGDIVVALGHTPVGSIFDLKVGSTKHTLQAIETRVLAGSKMTVARVVDPTPTPPP-E        2336
                          ** :: :.                       :: :.*   ::  :    ↑
```

```
gi|72|EAV_nsp7      PMPSRRR----------------RKGLPKG--AQLEWDRHQEEKRNAGDDDFAVSNDYVK    172
ABI64071.1          PMPSRRR----------------RKGLPKG--AQLEWDRHQEEKRNAGDDDFAVSNDYVK    1624
AEC48046.1          PVPED---------------AVTPIALENDARKLSRQDRDREE-IKRDSEKIGVVTVSGK    1984
AGA19089.1          TT--EHNGKKYLTVNGKTCFDHPQFKPEND-ARISKGTRDNEE-KKRDSEKLGSIVISGT    1986
YP_009109556.3      QVNGKFSPAKYLAVAGKVLADHPDYKLEND-GRFPRTR---ED-RVKDSVQVDTVDIGSH    2034
AZT89154.1          QVNGKFSPAKYLAVAGKVLADHPDYKLEND-GRFPRTR---ED-RVKDSVQVDTVDIGSH    2034
AAA74103.1          PSGL----------------V-----------KTSKKQARRQKRTGLGTEVVGTVVIDGV    2084
AAA85663.1          PSGL----------------V-----------KTSKKQRRRQKRTGLGTEVVGTVEIDGV    2064
AEJ54657.1          SIPL----------------QTPTPLFENGPRHRSEEDDLKVERMKKHCVSLGYHNINGK    2235
QPK93580.1          PVPI----------------PLPPRVLENGPNAGGEETVLNKKKR-RRMEAVGIYVMGGK    2248
ANT45956.1          PVPI----------------LLPPKVLENGPSAWGDENGLNKKKR-RRMEAVGIYVMGGK    2248
ALL55209.1          PVPI----------------PLPPKVLENGPRAWEDEDRLNKKRR-RKMEAVGIYVMDGK    2279
APU51031.1          PVPI----------------PLPPKVLENGPNAWGDEDRLNKKKR-RRMEALGIYVMGGK    2198
AFP43966.1          PVPI----------------PLPPKILENGPNAWGDEDRLNKKKR-RRMEAVGIFVMGGK    2379
                                                              .        .

gi|72|EAV_nsp7      RVPKYWDPSDTRGTTVKIAGTTYQKVVDYSGNVHYVEHQEDLLDYVL-------GKGSY-    224
ABI64071.1          RVPKYWDPSDTRGTTVKIAGTTYQKVVDYSGNVHYVEHQEDLLDYVL-------GKGSY-    1676
AEC48046.1          QYTKFWDKV-------------------TGDVWYSDEAAFP-------------------    2006
AGA19089.1          HYDKYWDKV-------------------SGDVWYEPITKESATPS---------------    2012
YP_009109556.3      TFKKMWNKT-------------------TGDVWYDIIMPESAANP---------------    2060
AZT89154.1          TFKKMWNKT-------------------TGDVWYDIIMPESAANP---------------    2060
AAA74103.1          SYNKVWHIA-------------------TGDVTYEGCLVTENPQLRP-LGM-TTIGRFQ    2122
AAA85663.1          SYNKVWHKA-------------------TGDVTYEGFLVSENSRLRT-LGT-SAIGRFQ    2102
AEJ54657.1          VYCKIWDKS-------------------TGDTFYTDDSRYTQDYAFQDRSADYRDGGY-    2274
QPK93580.1          KYQKFWDKN-------------------SGDVFYEEVHDNTDA-------------W-    2273
ANT45956.1          KYQKFWDKN-------------------SGDVFYEEVHDNTDA-------------W-    2273
ALL55209.1          KYQKFWDKN-------------------SGDVFYEEVHDNTDA-------------W-    2304
APU51031.1          KYQKFWDKN-------------------SGDVFYEEVHNNTDE-------------W-    2223
AFP43966.1          KYQKFWDKN-------------------SGDVFYEEVHDNTDA-------------W-    2404
                       * *.                      :*:. *

gi|72|EAV_nsp7      E-----------------------------------------------------------    225
ABI64071.1          EGLDQDKV----------------------------------------------------    1684
AEC48046.1          ------------------------------------------------------------    2006
AGA19089.1          ---------------------------------------------------------VQN    2015
YP_009109556.3      ------------------------------------------------------------    2060
AZT89154.1          ------------------------------------------------------------    2060
AAA74103.1          EFIRKHGEKVKTSVEKYPVGKKKSVEFNITTYL-LDGEEYDVPDHEPLEWTITIGESDLE    2181
AAA85663.1          EFIRKHGSKVKTSVEKYPVGKNKHIEFAVTTYN-LDGEEFDVPDHEPLEWTITIGDSDLE    2161
AEJ54657.1          EGVQTAPQHGFDPKSETPIGTVVIGGITYNRYLI-KGKEVLVPKP----------DNCLE    2323
QPK93580.1          ECLRTDDPTDLDPDRGTLCGHTTIDNKPYHVYAPPSGKKFLVPADP------KNVKAQWE    2327
ANT45956.1          ECLRADDSADLDPDRGTLCGHITIDNKPYHVYVSPSGKKFLVPANP------EDVKAQWE    2327
ALL55209.1          ECLRTDDPADLDPEKGTLCGRLIIENKPYCVYASPSGRKFLVPANP------ESGKAQWE    2358
APU51031.1          ECLRVGDPADFDPEKGTLCGHVTIENKAYHVYTSPSGKKFLVPVNP------ENGRVQWE    2277
AFP43966.1          ECLRVDNPADFDPEKGTLCGHTTIEDKAYSVYASPSGKKFLVPVNP------EGGRVQWE    2458
```

Supplementary Figure 21. Multiple sequence alignment of EAV nsp7. Within nidoviruses , EAV nsp7 aligns only with other arterivirus polyproteins. The arrow at EAV nsp7 residue 123 indicates the cleavage site utilized to separate nsp7α from nsp7β. Additional arrows indicate the locations of EAV nsp7 residues 143, 156, and 172 that were found to be modified by GMP. “*” indicates complete conservation, “:” indicates strong conservation, and “.” indicates weaker conservation per the Clustal Omega algorithm. The EAV nsp7 and EAV polyprotein (ABI64071.1) sequences are included as separate entries in the alignment.

Labeling compound	Mass addition	Mass Difference (reference = GTP)		
		z = 1	z = 2	z = 3
GTP	345.0474	-	-	-
^{15}N-GTP	350.0326	4.9852	2.4926	1.6617
^{13}C-GTP	355.0810	10.0336	5.0168	3.3445

Supplementary Table 1. Mass additions and differences for GMP, ^{15}N-GMP and ^{13}C-GMP-modification to peptides. The mass added to any peptide modified by GMP, ^{15}N-GMP and ^{13}C-GMP is shown as well as the mass differences between the GMP-labeled and the heavy-labeled GMP peptides for the specified charge states (z). Related to Figure 2c.

	c^+	Sequence	y^+	z^+	
1	75.05529	G			14
2	507.13475	S-Phosphoguanosine	1798.78635*	1783.77545*	13
3	635.22972	K	1366.70688*	1351.69598*	12
4	766.27020*	M	1238.61192*	1223.60102	11
5	853.30223*	S	1107.57144*	1092.56054*	10
6	968.32917*	D	1020.53941*	1005.52851*	9
7	1067.39759*	V	905.51247	890.50157*	8
8	1195.49255*	K	806.44405*	791.43315*	7
9	1355.52320*	C-Carbamidomethyl	678.34909	663.33819	6
10	1456.57088*	T	518.31844	503.30754	5
11	1543.60290*	S	417.27076	402.25986	4
12	1642.67132*	V	330.23873	315.22783	3
13	1741.73973*	V	231.17032*	216.15942	2
14		L	132.10191	117.09101	1

Supplementary Table 2. Ion series for GMP-modified SARS-CoV-2 nsp 7 pepitde 1-14. Calculated masses for c-, y-, and z-series ions that could be generated following EThcD fragmentation of nsp7 peptide 1-14 that is modified at serine-2. The ions, that were experimentally observed (+/- 0.04 Da) in the example spectrum from Figure 2d, are shown in red or blue coloring and are also denoted with an asterisk.

	b^+	Sequence	y^+	y^{2+}	
1	58.02874	G			17
2	171.11280 *	L	2309.04588	1155.02658	16
3	268.16557	P	2195.96182	1098.48455	15
4	741.30797	K-Phosphoguanosine	2098.90906	1049.95817	14
5	798.32943	G	1625.76666 *	813.38697 *	13
6	869.36654	A	1568.74519 *	784.87624 *	12
7	997.42512	Q	1497.70808 *	749.35768 *	11
8	1110.50918 *	L	1369.64950 *	685.32839 *	10
9	1239.55178 *	E	1256.56544 *	628.78636 *	9
10	1425.63109	W	1127.52285 *	564.26506 *	8
11	1540.65803	D	941.44353 *	471.22541	7
12	1696.75914	R	826.41659 *	413.71193 *	6
13	1833.81806	H	670.31548 *	335.66138	5
14	1961.87663	Q	533.25657 *	267.13192	4
15	2090.91923	E	405.19799 *	203.10263	3
16	2219.96182	E	276.15540 *	138.58134	2
17		K	147.11280	74.06004	1

Supplementary Table 3. Ion series for GMP-modified EAV nsp7 peptide 140-156. Calculated masses for b- and y-series ions that could be generated following HCD fragmentation of nsp7 peptide 140-156 that is modified at lysine-143. The ions, that were experimentally observed (+/- 0.04 Da) in the example spectrum from Figure 3a, are shown in red or blue coloring and are also denoted with an asterisk.

	b^+	Sequence	y^+	y^{2+}	
1	58.02874	G			17
2	171.11280*	L	2319.07945	1160.04336	16
3	268.16557	P	2205.99538	1103.50133	15
4	751.34153	K-Phosphoguanosine (13C)	2108.94262	1054.97495	14
5	808.36299	G	1625.76666*	813.38697*	13
6	879.40011	A	1568.74519*	784.87624*	12
7	1007.45869	Q	1497.70808	749.35768*	11
8	1120.54275	L	1369.64950*	685.32839*	10
9	1249.58534	E	1256.56544*	628.78636*	9
10	1435.66466	W	1127.52285*	564.26506*	8
11	1550.69160	D	941.44353*	471.22541	7
12	1706.79271	R	826.41659*	413.71193*	6
13	1843.85162	H	670.31548*	335.66138	5
14	1971.91020	Q	533.25657*	267.13192	4
15	2100.95279	E	405.19799*	203.10263	3
16	2229.99538	E	276.15540*	138.58134	2
17		K	147.11280	74.06004	1

Supplementary Table 4. Ion series for ^{13}C-GMP-modified EAV nsp7 peptide 140-156. Calculated masses for b- and y-series ions that could be generated following HCD fragmentation of nsp7 peptide 140-156 that is modified at lysine-143. The ions, that were experimentally observed (+/- 0.04 Da) in the example spectrum from Figure 3b, are shown in red or blue coloring and are also denoted with an asterisk.

	b^+	b^{2+}	Sequence	y^+	
1	157.10839*	79.05783	R		17
2	271.15131*	136.07930	N	2135.83643	16
3	342.18843	171.59785	A	2021.79350	15
4	399.20989*	200.10858	G	1950.75639	14
5	514.23684*	257.62206	D	1893.73493	13
6	629.26378*	315.13553	D	1778.70798	12
7	744.29072*	372.64900	D	1663.68104	11
8	891.35914*	446.18321	F	1548.65410	10
9	962.39625	481.70176*	A	1401.58568	9
10	1061.46466*	531.23597	V	1330.54857	8
11	1148.49669*	574.75198	S	1231.48016	7
12	1262.53962*	631.77345	N	1144.44813	6
13	1377.56656*	689.28692	D	1030.40520	5
14	1540.62989*	770.81858	Y	915.37826	4
15	1639.69830*	820.35279	V	752.31493	3
16	1767.79327*	884.40027	K	653.24652	2
17			R-Phosphoguanosine (15N)	525.15155	1

Supplementary Table 5. Ion series for ^{15}N-GMP-modified EAV nsp7 peptide 157-173. Calculated masses for b- and y-series ions that could be generated following HCD fragmentation of nsp7 peptide 157-173 that is modified at arginie-173. (Note that Sequest matched the GMP modification to arginine-173 instead of lysine-172 for this HCD spectrum) The ions, that were experimentally observed (+/- 0.04 Da) in the example spectrum from Figure 3c, are shown in red or blue coloring and are also denoted with an asterisk.

	c^+	$c+H^+$	Sequence	y^+	z^+	$z+H^+$	$z-H^+$	
1	75.05529	76.06311	G					14
2	468.11262	469.12044	S-PhosphoUridine	1759.76421*	1744.75332	1745.76114*	1743.74549	13
3	596.20758*	597.21541*	K	1366.70688	1351.69598	1352.70381*	1350.68816	12
4	727.24807*	728.25589*	M	1238.61192*	1223.60102*	1224.60885*	1222.59320*	11
5	814.28010*	815.28792*	S	1107.57144	1092.56054*	1093.56836	1091.55271*	10
6	929.30704*	930.31486*	D	1020.53941*	1005.52851*	1006.53633*	1004.52068*	9
7	1028.37545*	1029.38328*	V	905.51247	890.50157*	891.50939*	889.49374*	8
8	1156.47042*	1157.47824	K	806.44405*	791.43315*	792.44098	790.42533*	7
9	1316.50106*	1317.50889*	C-Carbamidomethyl	678.34909*	663.33819	664.34601	662.33036	6
10	1417.54874*	1418.55657*	T	518.31844*	503.30754*	504.31537	502.29972*	5
11	1504.58077*	1505.58860*	S	417.27076*	402.25986*	403.26769	401.25204*	4
12	1603.64918*	1604.65701*	V	330.23873	315.22783	316.23566	314.22001	3
13	1702.71760*	1703.72542*	V	231.17032*	216.15942	217.16725	215.15160	2
14			L	132.10191	117.09101	118.09883	116.08318	1

Supplementary Table 6. Ion series for UMP-modified SARS-CoV-2 nsp 7 pepitde 1-14. Calculated masses for c-, y-, and z-series ions that could be generated following EThcD fragmentation of nsp7 peptide 1-14 that is modified at serine-2. The ions, that were experimentally observed (+/- 0.04 Da) in the example spectrum from Figure 5e, are shown in red or blue coloring and are also denoted with an asterisk.
