# Supplementary material for: Mass spectrometric based detection of protein nucleotidylation in the RNA polymerase of SARS-CoV-2
Source: Commun Chem. 2021 Mar 19;4:41. doi: 10.1038/s42004-021-00476-4 (PMC8238455; doi:10.1038/s42004-021-00476-4)
Supplement: Supplementary file 2 — Description of Additional Supplementary Files [file 42004_2021_476_MOESM2_ESM.pdf]

Description of Additional Supplementary Files

File Name: Supplementary Data 1
Description: Data analysis of LC-MS peaks for a single SARS-CoV-2 chymotrypsin-digested sample that contained peptides labeled with GMP, ^{15}N-GMP and ^{13}C-GMP. "Search set up" tab shows formulas and criteria to search each LC-MS peak against all others ("index to search" tab). Two additional tabs show search results and filtered results.

File Name: Supplementary Data 2
Description: Data analysis of LC-MS peaks for a single SARS-CoV-2 GluC-digested sample that contained peptides labeled with GMP, ^{15}N-GMP and ^{13}C-GMP. "Search set up" tab shows formulas and criteria to search each LC-MS peak against all others ("index to search" tab). Two additional tabs show search results and filtered results.

File Name: Supplementary Data 3
Description: Data analysis of LC-MS peaks for EAV trypsin-digested samples that were not labeled with GMP or were labeled with either GMP, ^{15}N-GMP or ^{13}C-GMP. Peaks unique to two labeled, replicate injections (GMP-, ^{15}N-GMP or ^{13}C-GMP-labeled) were exported and searched against all others ("index to search" tab). Three tabs total.

File Name: Supplementary Data 4
Description: Summary of all MS/MS peptide spectrum matches containing a GMP, ^{15}N-GMP, or ^{13}C-GMP adduct. Top scoring hits for each peptide mass (m/z) and for each fragmentation method (HCD or EThcD) are shown along with peptide sequence, Xcorrelation score, precursor mass error, raw data file name, scan number, and other relevant information.
