# Supplementary material for: Mass spectrometric based detection of protein nucleotidylation in the RNA polymerase of SARS-CoV-2
Source: Commun Chem. 2021 Mar 19;4:41. doi: 10.1038/s42004-021-00476-4 (PMC8238455; doi:10.1038/s42004-021-00476-4)
Supplement: Supplementary file 8 — Reporting Summary [file 42004_2021_476_MOESM8_ESM.pdf]

nature research

Corresponding author(s): Michael R. Sussman

Last updated by author(s): 02/16/2021

Reporting Summary

Nature Research wishes to improve the reproducibility of the work that we publish. This form provides structure for consistency and transparency in reporting. For further information on Nature Research policies, see our Editorial Policies and the Editorial Policy Checklist.

Statistics

For all statistical analyses, confirm that the following items are present in the figure legend, table legend, main text, or Methods section.

n/a	Confirmed	
☐	☒	The exact sample size (n) for each experimental group/condition, given as a discrete number and unit of measurement
☐	☒	A statement on whether measurements were taken from distinct samples or whether the same sample was measured repeatedly
☒	☐	The statistical test(s) used AND whether they are one- or two-sided<br>*Only common tests should be described solely by name; describe more complex techniques in the Methods section.*
☒	☐	A description of all covariates tested
☒	☐	A description of any assumptions or corrections, such as tests of normality and adjustment for multiple comparisons
☒	☐	A full description of the statistical parameters including central tendency (e.g. means) or other basic estimates (e.g. regression coefficient) AND variation (e.g. standard deviation) or associated estimates of uncertainty (e.g. confidence intervals)
☒	☐	For null hypothesis testing, the test statistic (e.g. F, t, r) with confidence intervals, effect sizes, degrees of freedom and P value noted<br>*Give P values as exact values whenever suitable.*
☒	☐	For Bayesian analysis, information on the choice of priors and Markov chain Monte Carlo settings
☒	☐	For hierarchical and complex designs, identification of the appropriate level for tests and full reporting of outcomes
☒	☐	Estimates of effect sizes (e.g. Cohen's d, Pearson's r), indicating how they were calculated

Our web collection on statistics for biologists contains articles on many of the points above.

Software and code

Policy information about availability of computer code

Data collection	Mass spectrometry data was acquired using Thermo Scientific Xcalibur software version 4.3.73.11.
Data analysis	Data was analyzed with Thermo Proteome Discoverer version 2.4.1.15, Microsoft Excel Version 2006 (Build 13001.20384) and Thermo Scientific Freestyle software version 1.5.93.34.

For manuscripts utilizing custom algorithms or software that are central to the research but not yet described in published literature, software must be made available to editors and reviewers. We strongly encourage code deposition in a community repository (e.g. GitHub). See the Nature Research guidelines for submitting code & software for further information.

Data

Policy information about availability of data

All manuscripts must include a data availability statement. This statement should provide the following information, where applicable:

- Accession codes, unique identifiers, or web links for publicly available datasets
- A list of figures that have associated raw data
- A description of any restrictions on data availability

The raw mass spectrometry datasets and proteome discoverer results that were generated and analyzed in the current study are available through the MassIVE repository under the accession / identifier MSV000085857 [https://doi.org/doi:10.25345/C52B2B]. All figures were derived from this data with the exception of Figure 1, Supplementary Figure 1, Supplementary Figure 12-14 and Supplementary Figures 16 - 21. Data is available to the public.

Field-specific reporting

Please select the one below that is the best fit for your research. If you are not sure, read the appropriate sections before making your selection.

- [x] Life sciences
- [ ] Behavioural & social sciences
- [ ] Ecological, evolutionary & environmental sciences

For a reference copy of the document with all sections, see nature.com/documents/nr-reporting-summary-flat.pdf

Life sciences study design

All studies must disclose on these points even when the disclosure is negative.

Sample size

No sample-size calculation was performed. Initial results demonstrated strictly unambiguous outcomes, i.e. band radiolabeling was readily apparent from background and/or non-specific signals. Because we did not need quantitation in our study to determine positive results, we deemed sample sizes greater than n=1 where not needed in a single experiment, and instead reasoned that replication of the experiment multiple times on different days suited the experimental purposes sufficiently.

Data exclusions

No data was excluded from analyses.

Replication

Gel-based nucleotidylation reactions that included minimally WT EAV nsp9 and nsp7 or SARS-CoV-2 nsp12, nsp7 and nsp8 were repeated at least three times, though not with the identical set-up as displayed in Figure 1. All attempts at replication were successful. The mass spectrometry assay directly supports the gel-based assay, so additional replication was deemed unnecessary.

For mass spectrometry analysis, a single experiment provided the initial, reported nucleotidylated data for theEAV and SARS-CoV-2. Technical replicates were performed either simultaneously (EAV) or on a different day (SARS-CoV-2) and yielded the same results (ie presence of appropriate m/z in appropriate samples). Biological replicates (separate experiments) for modification of WT proteins were performed in the more advanced experimental set-up examining the mutant proteins described in the text. Results confirmed the initial dataset but are not directly reported, although the data is available in the repository described above.

LC-MS/MS examination of GMP-labeling of mutant proteins SARS-CoV-2 nsp7 S2A, SARS-CoV-2 nsp7 K3A, EAV nsp9 K380A and EAV nsp7 K156A was performed in a single experiment. All other mutants discussed were examined in two separate experiments. No data was omitted. Though these experiments looked for the disappearance of GMP-labeling on the mutated residue (i.e. negative data), success of the experiment was gauged by verifying GMP-labeling of a WT control in every new experiment and verifying GMP-labeling of other known sites within the mutant protein samples. Radiolabeling of EAV mutant proteins were performed once and verified with LC-MS/MS, whereas the SARS-CoV-2 mutant radiolabeling was performed twice in separate experiments, which was verified with LC-MS/MS in additional experiments.

Further biological replicates were not performed because 1) the gel based assay supported the mass spectrometry data, 2) the experimental design incorporated controls (ie unlabeled, GTP-labeled, 15N-GTP-labeld and 13C labeled samples) that generated mathematically-predictable and very specific results without ambiguity, 3) the nature of LC-MS/MS itself (even without the internal controls) is highly precise and accurate such that errors are highly unlikely given the stringent matching criteria (precursor mass errors < 1.5 ppm, MS/MS fragment ion detection in orbitrap with 0.04 Da error tolerance, high Sequest Xcorrelation scores for top peptide spectrum matches that are > 2.5, and false-discovery rate less than 1%), and 4) the analyses generated unique but redundant results for many peptides (e.g. the SARS-CoV-2 GMP modification was observed in four different peptides in MS/MS).

Estimates of percent protein labeling with radionucleotide was performed once as an estimate and for additional information requested by reviewers. Because this data was not central to the conclusions of the study, it was only performed once.

Competition of radiolableing with cold nucleotide was performed in three separate experiments with consistent results. Again, because data was not central to the conclusions of the study, no further replication was performed.

Radiolabeling with UMP was performed in a single experiment with multiple technical replicates. Because this data was followed up by more informative experiments via LC-MS/MS, we did not performed additional UMP radiolabeling experiments.

LC-MS/MS data of UMP-peptide adducts were collected from a single biological experiment. Two replicates were examined. Further replication was not performed due to the same reasons provided above for LC-MS/MS data and because our study ended with this data that was central to the main conclusions and results of this paper.

Randomization

The experimental samples were varied by a single factor (no nucleotide, GTP, 15N-GTP, 13C-GTP, etc.) in a very quick 30 minute assay such that covariates were not considered. Randomization was not performed. Injection order onto the LC-MS/MS generally started with negative controls first (ie "no nucleotide" reactions) to minimize carryover. Between batches of analyses, the LC columns were washed extensively and monitored for unacceptable levels of contamination. Initial analyses also assessed carryover from prior samples; we determined the carryover was very low and did not obscure data interpretation.

Blinding

Blinding was not applied for the following reasons. The gels were generally loaded in a specific order that facilitated experimental result interpretation. In addition, successful nucleotidyation radiolabeling was readily distinguished from non-specific background, which did not require a scoring regime. For the mass spectrometry data, the raw data needed to be subjected to a complicated analysis after acquisition

that required knowing the exact identity of the samples (e.g. unlabeled, GTP-labeled, 15N-GTP-labeld, 13C labeled, etc). In addition, the applied analyses were defined in a purely mathematical manner and do not rely on any subjective scoring regime.

Reporting for specific materials, systems and methods

We require information from authors about some types of materials, experimental systems and methods used in many studies. Here, indicate whether each material, system or method listed is relevant to your study. If you are not sure if a list item applies to your research, read the appropriate section before selecting a response.

Materials & experimental systems

n/a	Involved in the study
☒	☐ Antibodies
☒	☐ Eukaryotic cell lines
☒	☐ Palaeontology and archaeology
☒	☐ Animals and other organisms
☒	☐ Human research participants
☒	☐ Clinical data
☒	☐ Dual use research of concern

Methods

n/a	Involved in the study
☒	☐ ChIP-seq
☒	☐ Flow cytometry
☒	☐ MRI-based neuroimaging
